# Subliminal Salience Search Illustrated: EEG Identity and Deception Detection on the Fringe of Awareness

**DOI:** 10.1371/journal.pone.0054258

**Published:** 2013-01-23

**Authors:** Howard Bowman, Marco Filetti, Dirk Janssen, Li Su, Abdulmajeed Alsufyani, Brad Wyble

**Affiliations:** 1 Centre for Cognitive Neuroscience and Cognitive Systems (CCNCS), School of Computing, University of Kent, Canterbury, Kent, United Kingdom; 2 NHTV Breda University of Applied Sciences, Breda, The Netherlands; 3 Experimental Psychology, Cambridge University, Cambridge, United Kingdom; 4 Department of Psychology, The College of Arts and Sciences, Syracuse University, Syracuse, New York, United States of America; University of Buenos Aires, Argentina

## Abstract

We propose a novel deception detection system based on Rapid Serial Visual Presentation (RSVP). One motivation for the new method is to present stimuli on the fringe of awareness, such that it is more difficult for deceivers to confound the deception test using countermeasures. The proposed system is able to detect identity deception (by using the first names of participants) with a 100% hit rate (at an alpha level of 0.05). To achieve this, we extended the classic Event-Related Potential (ERP) techniques (such as peak-to-peak) by applying Randomisation, a form of Monte Carlo resampling, which we used to detect deception at an individual level. In order to make the deployment of the system simple and rapid, we utilised data from three electrodes only: Fz, Cz and Pz. We then combined data from the three electrodes using Fisher's method so that each participant was assigned a single p-value, which represents the combined probability that a specific participant was being deceptive. We also present subliminal salience search as a general method to determine what participants find salient by detecting breakthrough into conscious awareness using EEG.

## Introduction

### 1.1 Subliminal Salience Search

Rapid serial visual presentation (RSVP) [1], [2] and associated electrophysiological components [3], [4] have been extensively used in the theoretical study of attention, perception, consciousness and working memory. In particular, a set of key theoretical phenomena have been identified using RSVP, e.g. the attentional blink [5]–[8], repetition blindness [9], [10] temporal conjunction errors [11], [12], conceptual short term memory [2], spreading the sparing [13] and contingent capture [14]. However, the *practical* application of this presentation format, especially when combined with EEG, has had very little exposure. An early consideration of how RSVP-EEG might be applied is a technical report on single trial P3 detection and human-computer interaction [15]. Furthering the practical application of such methods is the main objective of this paper.

RSVP reveals an extraordinary perceptual capacity of the human cognitive system. Stimuli are presented at around 10 per second (and sometimes as fast as 20 per second [5]) each replacing its predecessor at the same spatial location and the participant is usually tasked with detecting or identifying a target. For example, the task might be to report the identity of the sole letter (the target) in a stream of digits (the distractors). At RSVP rates, capacity to identify a single target within a stream of non-target (distractor) items, can be as high as 90% and is rarely below 70%. Furthermore, performance is high across a broad variety of stimulus and task types, e.g. identifying a target letter in a stream of distractor digits [5], [6]; identifying the sole job word in a stream of nature word distractors [16]; identifying a stimulus marked by unique colour [11]; reading sentences with its words presented as RSVP frames [17]; and reporting the presence of a categorically specified picture, e.g. whether a target image of “dinner food” is present in a stream of pictures [18].

**Figure 1:**
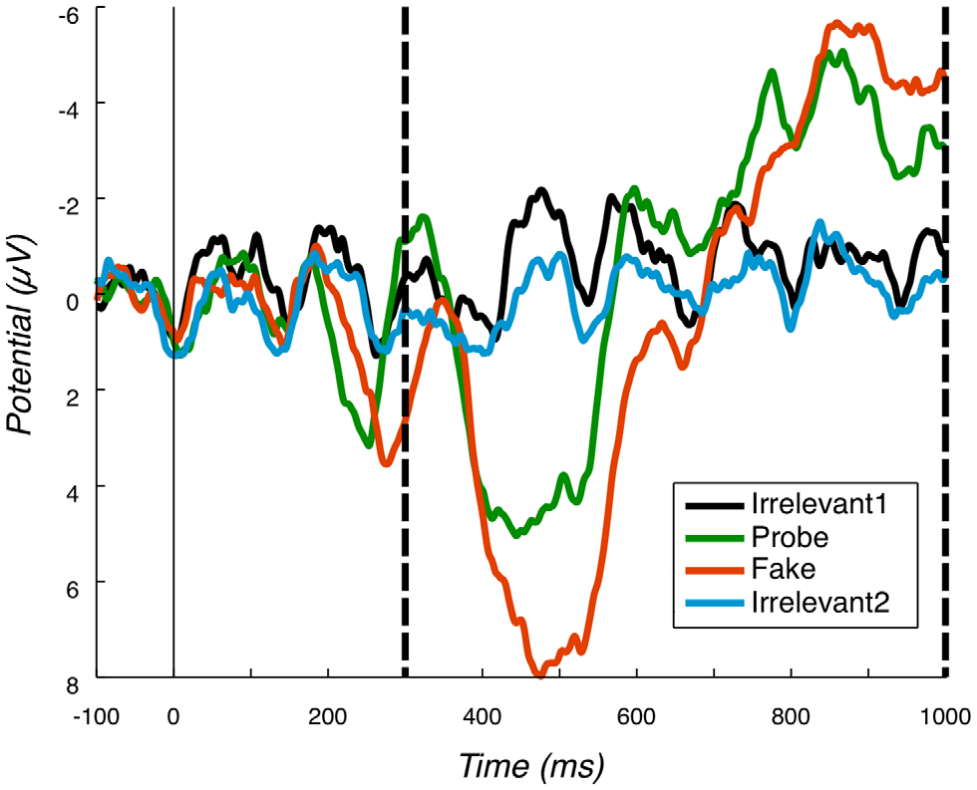
Pz grand average. Grand average at Pz, for all conditions. Positive is plotted down; y-axis is microvolts; x-axis is milliseconds. Vertical dashed lines mark the P3b bounding window. The amplitude of Probe and Fake is clearly larger than the amplitude of the two Irrelevant conditions. Moreover, the peak of Probe is earlier than the peak of Fake.

In addition, an electrophysiological marker of item perception in RSVP has been identified; that is, when an item is ‘seen’ in RSVP, a P3 locked to that stimulus presentation is generated. In contrast, ‘unseen’ items, typically distractors or missed targets, do not evoke the P3 component [15], [19], e.g. compare the P3b for Fake (seen target), with P3b for Irrelevant1 (unseen) in Figure 1.

These theoretically identified characteristics of RSVP make it, we contend, singularly suitable for a class of applications that could be called *subliminal salience search*. The term *search* is used since rapid streams of stimuli are presented and the participant's perceptual system searches amongst them. Conceptually, one could think that a participant's perceptual system is searching for an item matching one of a set of templates that a participant's cognitive system possesses. Indeed, due to the rapid presentation rate, the information bandwidth of the search is potentially very high. We use the term *salience*, since the participant's perceptual system is searching for a salient item. If you like, the templates the brain is seeking to match reflect what the participant's cognitive system finds salient. They are, effectively, salience templates. This salience could be strictly intrinsic and thus, incidental to the current task (e.g. one's own name or an inherently threatening stimulus while searching for a job-related word) or it could be prescribed by the current task (e.g. a hooded figure when searching a stream of CCTV images for a felon).

For example, in the context of an identity detection experiment, a stream of names would be visually presented, one of which might be the participant's real name and another a name they are pretending is theirs. Now, the brain will contain a template for their real name; this might, for example, comprise an assembly of neurons configured to detect the visual form of that name. In the context of this paper, such a real name template is considered *intrinsic*; that is, it is not specifically set-up from instructions of the current experiment; it has arisen from a life's experience of producing and comprehending one's own name. In contrast, a template for a pretend name would be specifically set-up under the task's instructions, yielding what one might call a task template.

The brain then “searches” amongst the names presented (in RSVP) to it for one matching one of its (presumably visual) templates. Specifically, this search could be expected to involve the brain's object recognition system, which is believed to be performed by the ventral visual processing (or what) pathway [20]. Thus, the brain is performing an, if you like, automatic and highly efficient pattern recognition, where the patterns being searched for reflect its salience templates.

The search system we consider is also, in a specific sense, *subliminal*. In RSVP, the vast majority of presented items are not consciously perceived by the participant, however, the participant's perceptual system must be sub-consciously comparing a large number of these items against the brain's salience templates. Indeed, if we assume, say, an 85% accuracy in reporting the single target in an RSVP stream, the perceptual system must be processing items sufficiently to make a salience decision on, at the least, 85% of the presented stimuli (modulo some correction for lucky guesses). However, typically, most of those stimuli will not be distinctly recognized at a conscious level, despite having been analysed for salience. For instance, it is invariably the case that few, if any, of the distractors in an RSVP stream are reportable.

In this respect, and putting many philosophy of consciousness issues aside, our criteria for judging a stimulus as subliminal, is its non-reportability at the end of an RSVP stream. The interaction between this subliminal search and supraliminal control is also notable. In particular, participants can volitionally impose a task template upon what is, we are arguing, a subliminal search. That is, conscious cognitive control can ‘reach down’ to set a task template, which is then applied subliminally.

The final ingredient of the proposed cognitive search system is *EEG-marking*. Our theoretical interpretation is that, during RSVP, the participant's brain is perceptually comparing fleetingly represented stimuli against its salience templates, until it detects a match. At that point, an attentional enhancement is applied, which typically propels that salient stimulus into conscious awareness, generating a clearly remembered percept of the stimulus. (Thus, in the context of perception in RSVP, we view attention as a mechanism, which facilitates conscious perception. In this sense, attention and consciousness are distinguished.) This processing sketch is consistent with the major RSVP models, e.g. [5], [10], [12], [21]–[24]. In addition, it seems that this ‘bursting into awareness’ generates a P3 event related potential component. Thus, in RSVP, not only is the brain searching for salient stimuli at very high presentation loads, we also have an electrophysiological marker of when it detects such a salient stimulus. These characteristics have led us to call the system we propose, *EEG-marked subliminal salience search (eegSSS)*.

There are many potential applications of the eegSSS method. We briefly highlight examples here, while considering some more fully in section 4.8. Typical applications would include, a brainwave acknowledgement system in human-computer interaction [15]; an independent brain-computer interface [25]; an image triage system [26]; information retrieval and stimulus-rich information presentation [27] and deception detecting. As a particularly compelling and emotive illustration of eegSSS, we focus on the latter here.

### 1.2 Deception detection

EEG-based lie detection has been extensively investigated using the P3 oddball paradigm, e.g. [28]. Many variations on this method have been proposed, e.g. [29]–[31], however all are confounded or, at the least, significantly complicated, by the possibility to consciously apply countermeasures. The main guilt determining comparison in these approaches is between the P3 response on *Probe* trials and on *Irrelevant* trials. Note that we use the term ‘guilt’ just to indicate the presence of concealed information, without implying the presence of a feeling or emotion of guilt. The guilty (concealed) knowledge is presented on Probe trials, while on Irrelevant trials, guilt- (indeed, task-) irrelevant stimuli are presented. (Note, *Target* trials are also typically presented, on which the task requires participants to falsely assert knowledge of an in fact irrelevant stimulus, but guilt can be judged without considering *Targets*.) The main lie detector confounding countermeasure involves participants artificially simulating a high salience brain response during Irrelevant trials, e.g. imagining the experimenter hitting them [32]. The aim of this countermeasure is to increase the size of the Irrelevant P3, such that it becomes statistically indistinguishable from the Probe P3.

Lie detecting on the fringe of awareness offers the potential to subvert this countermeasure. By presenting Irrelevant stimuli below the threshold of awareness, a volitional strategy to heighten the response selectively to Irrelevants is itself countered. An RSVP-based eegSSS is a natural method to realise such a fringe awareness deception detector. Specifically, stimuli can be presented in RSVP format (around 10 items a second), such that a single *critical* item (i.e. that plays a role in the guilt detection task) is present on each trial, i.e. a Probe, an Irrelevant or a Fake. Note that in our paradigm, we use the term *Fake*, rather than *Target*, because our participants actively select their *Fake* name. In some of the alternative paradigms, e.g. [33], *Targets* are otherwise irrelevant stimuli with a particular target-defining characteristic (e.g. a specific colour or font). The presence of the *Fake* is crucial in our paradigm, as it is enforces the task set that participants must follow (they will be asked to report its presence after each RSVP trial; see section 2.3); this forces them to attend the stream.

Our hypothesis is that participants' perceptual systems will amplify salient items. This will enhance the brain's representation of the Fake and, for the guilty, the Probe, while not for stimuli that are non-salient, i.e. always the Irrelevant and, for the nonguilty, the Probe. In particular, since the choice of stimulus to act as Irrelevant is unknown to the participant, it should be possible to repeat RSVP trials containing the Irrelevant, with little if any, participant awareness of their presence. If participants, indeed, remain unaware of Irrelevant stimuli, their electrophysiological response, i.e. their P3, should remain small or, even, absent. In contrast, the P3 evoked by the (intrinsically salient) Probe should be large, as a reflection of enhancement by the brain and consequent perceptual breakthrough.

Importantly, in respect of possible eegSSS applications, the deception detector is particularly reliant on observing an electrophysiological response to *intrinsic* salience. That is, as already indicated, a stimulus may be salient either because it conforms to a currently configured task template (e.g. ‘cook’ when looking for job words) or because it is intrinsically salient (e.g. one's own name when searching for job words). In the theoretical RSVP literature, most studies have considered (non-intrinsic) task-specified search. These studies have demonstrated large behavioural responses to task-specified targets, e.g. deep first-target evoked attentional blinks [6]. In contrast, behavioural responses to intrinsically salient stimuli have often been small, e.g. attentional blinks evoked by threatening stimuli [34]. The size, scalp topography and latency of the RSVP P3 evoked by intrinsically salient stimuli is an empirical question, which we will throw light on in this paper.

### 1.3 Components and analysis methods

Due to the methodological requirements associated with detecting P3 deflections in RSVP deception detection, we have employed a number of analysis methods not typically found in the event related potential literature. A particular challenge is that sufficient statistical power needs to be available for *individuals* to be demonstrated as deceiving, i.e. solely on the basis of their data. Thus, the statistical bar is, in general, higher for a deception detector than a typical theoretical ERP study, where group-level significance is sufficient. Furthermore, this necessity to demonstrate individual-level significance, requires statistical inference not reliant upon quantifying variance across participants. An individual's error variance can, though, be deduced using Monte Carlo random resampling techniques, such as bootstrapping and randomisation [35]. Previous ERP lie detectors have employed bootstrapping, e.g. [28]. We, however, apply randomisation which, when sufficient resamplings are taken, accurately approximates exhaustive permutation tests, which are, in a specific sense, statistically exact; see p. 15 and 16 of [35]. Randomisation tests are used extensively to analyse fMRI data [36] and have, indeed, been successfully applied to EEG data [37], [38].

We will focus on two P3 variants: the P3a and the P3b. The P3a is classically observed earlier and with a more frontal distribution: it is typically maximal over Fz (Frontal midline) and Cz (Central midline, i.e. vertex). In contrast, the P3b peaks later and is maximal over Pz (Parietal midline). The P3a is classically elicited by a task-irrelevant oddball (hence, the commonly used name “novelty P3”), while the P3b is elicited by a task-relevant oddball [39]. Pre-empting our findings somewhat, while indeed observed frontally, the early component we will observe, will occur somewhat before the P3a's highlighted in the literature. Nonetheless, we believe the term P3a is appropriate and we justify this identification in the discussion, where we also detail why we observe both a P3a and P3b.

**Figure 2:**
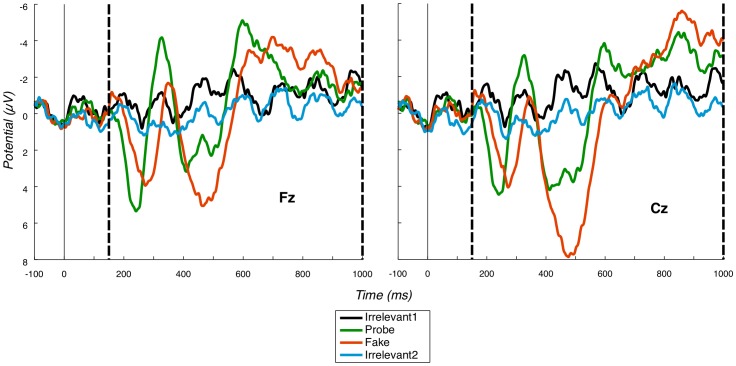
Fz and Cz grand averages. P3a grand averages at Fz and Cz, positive down, vertical dashed lines mark the region in which we search for the P3a (i.e. the bounding window). There is evidence of a large P3a for the Probe condition. Probe is also earlier than Fake.

To accommodate individual differences in P3 latency and form, we directly search for both P3a and P3b for each participant. Specifically, our P3a component typically manifests as a full oscillation cycle, with a sharp positive and then negative deflection (Figure 2). Our P3b again manifests as a positive followed by negative, although the P3b's negative deflection is much more temporally smeared than the positive deflection. To reflect this biphasic pattern, we perform a peak-to-peak analysis, as advocated by Rosenfeld and coworkers, e.g. [40]. This entails searching to find peaks in ERPs and, then, measuring the *maximum* change in voltage between highest (typically positive) and lowest (typically negative) peaks. To robustly calculate a probability of familywise Type I error in our per-participant randomisation analysis, as detailed shortly, we apply the same peak identification method (taking the *maximum* peak-to-peak difference) on each random resampling. In particular, such *maximal* value randomisation automatically controls for multiple comparisons in possible window placements.

In addition, we explore combining P3a and P3b tests into a single joint statistical inference. This enables us to aggregate statistical significance across measures on three different dimensions: P3a-Fz, P3a-Cz and P3b-Pz. To make the measures comparable, we employed a Fisher combined probability test, as used for example, to combine cluster size and voxel intensity in fMRI randomisation analyses [41]. This set-up has the advantage of being easily implemented: only three electrodes (Fz, Cz and Pz) carry the information needed in our analysis. We test in simulation the false positive rate of our analysis method, assuring its intrinsic validity. Specifically, we build a null dataset from segments of our EEG uncontaminated by Probe or Fake, such that, the null hypothesis is by construction true. These simulations demonstrate that when the null hypothesis is true, our randomisation method, including the Fisher combined analysis, yields the expected false positive rate of 0.05, i.e. the alpha level.

Using many of these same basic measures, we also undertake group-level significance analysis. The participant-specific identification of peaks yields a group-level analysis in which some parameter settings are treated as random effects. That is, rather than being fixed across all participants (e.g. from the grand average), positions of peaks vary from individual to individual.

We also run two other group-level analyses. Our main comparisons of Probe against Irrelevants suggest that Irrelevants are not robustly perceived. However, to obtain further evidence of Irrelevant's imperceptibility, which is key to our proposal, we seek to determine whether the repeated presentation of Irrelevants could influence their electrophysiological response. If ‘unknown’ stimuli could be ‘noticed’ because they appeared frequently, we should see a larger P3 later in the experiment (when they had indeed been previously presented often). Accordingly, we compare ERPs for Irrelevants between early and late in the experiment. Finally, we are also interested to demonstrate that Fake and Probe conditions are different, since this would indicate that the response we observe for Probes is differentiable from pure task-oriented target detection, which would underlie the Fake ERP. Consequently, we calculate the difference in P3 latency between the Fake and Probe conditions and, thereby, verify a difference in temporal features between the two.

## Methods

### 2.1 Participants

Fifteen participants undertook the experiment; all were students at the University of Kent, in the age group 18–24 (*M*: 20.47, *SD*: 2.53). All were paid for participating. All participants were right handed; 9 female, 6 male. Participants were free from neurological disorders and had normal or corrected-to-normal vision. Only native English speakers participated in the experiment.

#### 2.1.1 Ethics

This study was approved by the University of Kent Psychology Ethics Committee, which follows the guidelines set by the British Psychological Society regarding experiments with human participants. The study was approved as reference number 20101504. Written consent was obtained from all participants.

### 2.2 Stimulus Presentation

**Figure 3:**
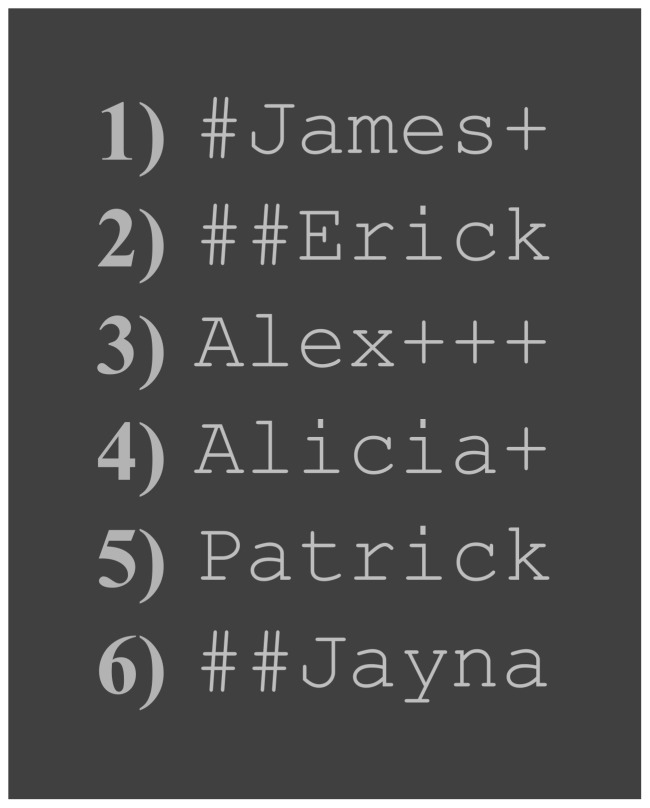
Example stimuli. List of example names, formatted as stimuli. Note that name 3 would not be shown immediately after name 4 as they have 2 letters (‘A’ and ‘L’) in the same position.

We presented RSVP streams on a 20″ LCD screen with a refresh rate of 60 Hz and a resolution of 1600×1200, placed at a distance of 60 cm from the participant. We used custom scripts that employed the Psychophysics toolbox version 3, running under Matlab 2010a. Stimuli were 16 point, light grey (75% white; RGB:190,190,190) monospaced, sans-serif characters presented on a dark (25% white; RGB:64,64,64) background. As a result, the visual angle for each stimulus was 0.48° in height and 2.48° in width, whereas the whole screen consisted of a rectangle of 28.52° by 37.56°. The Stimulus Onset Asynchrony (SOA) was 133 ms. Each RSVP trial consisted of a stream of 15 items, plus a starting and finishing item. The starting item was XXXXXXX, presented for 800 ms, in order to position participant's focus on the stimulus presentation area. The finishing item was either ------- or  =  =  =  =  =  =  = , selected at random, and remaining on screen for 133 ms. The response phase began by asking the participant to identify the finishing item. We used this to keep attention focused on the stream after the critical item (Probe, Fake or Irrelevant1/2) had been presented, thereby avoiding muscle artefacts caused by response preparation and initiation before stream end. Apart from starting and finishing items, all stimuli were common English proper names with a maximum length of 7 characters, and first letter capitalised. We padded shorter names using a randomising algorithm, with ‘#’ or ‘+’ characters blocked on each side of the word (Figure 3). Distractor names were chosen pseudorandomly: in order to avoid repetition, names could not contain two or more letters in the same position as their immediate predecessor. In addition, names which shared three or more letters in the same position as one of the critical items were not presented as distractors. We presented all stream items at the same screen location.

### 2.3 Stimuli

As previously indicated, we call *Irrelevant1*, *Irrelevant2*, *Probe* or *Fake* stimuli *critical* items. These critical items could be the participant's real name (*Probe*), their assumed name (*Fake*) or one of two preselected names, unknown to the participant (*Irrelevant1* or *Irrelevant2*). There were 3 blocks, each consisting of a random sequence of *Irrelevant1*, *Irrelevant2*, *Probe* and *Fake* trials. For each trial type, there were 50 RSVP trials. Each trial of 15 items contained only one critical item and 14 randomly chosen names as distractors. The position of the critical item within the stream was selected pseudorandomly, so that it had equal probability of appearing in the 5^th^ position (earliest) through to the 10^th^ position (latest).

We generated a set of possible names from the USA Social Security Administration database (http://www.ssa.gov/oact/babynames/). The 1000 top names from four different years (2009, 1969, 1929 and 1890) were combined into a single set of unique names. We only kept names shorter than 8 characters, resulting in a total set size of 3667 names. Prior to the start of the experiment, we presented participants with a subset of 12 possible female or male names, depending on their gender, from which they removed all names of people they knew well. Participants then chose one of the remaining names as their *Fake* name. After each RSVP stream, they were asked, on-screen, “did you see your name”? We had previously instructed participants to answer “Yes” if they had seen their Fake name and “No” otherwise, including when they saw their real name (the *Probe*) (participants' responses to this question are reported in Table 1). We chose two further names unfamiliar to the participant from the subset of twelve possible names and used them as *Irrelevant1* and *Irrelevant2*. Experimentally, we treated these identically; their only difference was in the (random) choice of name. Furthermore, Irrelevants were identical to distractors apart from the frequency with which they occurred over the course of the experiment (50 times each and approximately once per distractor).

**Table 1:** Number of times that “Yes” was answered at the end of each trial type.

Part. No.	Fake	Probe	Irrelevant1	Irrelevant2
1	48	1	2	1
2	37	1	1	3
3	48	0	5	3
4	50	0	0	1
5	50	0	1	0
6	40	3	0	2
7	49	0	4	5
8	50	0	0	0
9	50	1	2	1
10	46	0	1	6
11	42	3	5	1
12	47	2	1	1
13	48	2	5	0
14	46	0	1	0
15	50	0	5	2

The number of times that a positive answer was given to the “Did you see your name?” question is reported in this table, for each trial type. All participants followed our instructions correctly, responding “Yes” almost exclusively after trials that contained their *Fake* name.

### 2.4 Recording Apparatus

We recorded data using a Brain Products QuickAmp recorder (BrainProducts, Munich, Germany). We bandpass filtered data at recording, with a low-pass of 85 Hz and a high-pass of 0.30 Hz. We recorded Electroencephalographic data from the Fz, Cz, P3, Pz, P4, A1 and A2 electrodes using the standard 10–20 system (Jasper, 1958). We recorded electrooculograms from the left and right eyes using two bipolar HEOG and VEOG electrodes. During recording, we used the average of all channels as reference (common reference). We kept impedances below 7 kOhm (2.27 kOhm on average).

### 2.5 Analysis Procedure

We analysed data with Brain Products Brain Vision Analyzer version 1.05. At analysis, we software filtered data with a low-pass of 45 Hz and high-pass of 0.5 Hz, with a slope of 12 dB. We also applied a notch filter at 50 Hz to remove any potential electrical interference. We re-referenced data to the average of the combined mastoids (electrodes A1 and A2). We detected eye blinks using the “Gratton & Coles” algorithm [42] in Analyzer and every trial that contained an eye blink marker was excluded from the remaining analysis. Trials were visually inspected so that any trial containing electrical activity below −50 µV or above +50 µV was rejected. For further analyses, we used EEGLAB version 9 under Matlab 2010a [43]. We calculated ERPs using −100 ms to 1000 ms stimulus-locked windows, baseline corrected from −100 ms to 0 ms.

### 2.6 P3 differences

For each condition (Probe, Fake and Irrelevant2), we estimate three different P3 measures, named P3b-Pz, P3a-Fz and P3a-Cz. This is done on a participant-by-participant basis (on participant-level ERPs). These three measurements are determined from the point-wise difference between the ERP of the given condition and the ERP of the Irrelevant1 condition, which plays the role of baseline. The measure employed is the peak-to-peak value of the difference wave (condition minus Irrelevant1). In more detail, initially, the raw difference between the ERP of the given condition and the ERP of the Irrelevant1 condition is calculated. The result of this operation is a difference wave, which in certain conditions contains a P3 signal. In order to determine the intensity of the signal, a peak-to-peak measurement procedure is applied to this difference wave. Two parameters of this procedure vary depending upon the channel: P3b parameters are applied at Pz, P3a parameters at Fz and Cz. The first parameter is the start of the time window in which we *search* for the P3 (strictly, search for its highest and lowest peaks), we call this the *bounding* P3 window. For the P3b, the bounding window starts at 300 ms from target onset and ends at 1000 ms from target onset, whereas for the P3a the bounding window starts at 150 ms (and still ends at 1000 ms). We consider the extent and placement of these bounding windows to be a priori justified by the P3 literature and thus not subject to multiple comparison's correction [44]. The second parameter that varies between P3b and P3a analysis is the presence of a boundary that limits the search for the highest peak, which is present only for the P3a analysis (this is discussed in more detail in the next section).

### 2.7 Peak-to-Peak

The peak-to-peak procedure we applied to the (Condition minus Irrelevant1) difference waves determines the disparity between the highest peak and the lowest (following) peak in the specified P3 bounding window. Note that peaks here are not in fact single time points, but rather averages across relatively small windows of time points. This usage is consistent with peak-to-peak measurements used in previous P3 deception detection research [31]. (For the purpose of this paper, the word peak will always refer to such an average). Hence, peaks were identified as the highest or lowest averages across inner windows of 100 ms, i.e. each peak corresponds to the mean voltage of that window. (We use the term *inner* window to refer to a time interval across which we calculate the average amplitude.) The procedure finds the highest peak first, by iterating through all 100 ms (inner window) intervals from the start of the P3 bounding window until its end. In other words, we slide a 100 ms interval across the bounding window, looking for the interval with the highest average. For the P3a, the search for the highest peak ends at 300 ms from critical item onset. The presence of this boundary prevents the P3b (whose start was previously pinpointed at 300 ms in RSVP experiments [45]) from being detected as the highest peak of the P3a. After the highest peak is found, the procedure then continues iterating from the first non-overlapping position that followed the highest peak until the end of the P3 bounding window, searching for the lowest peak. The peak-to-peak measurement is finally calculated as highest minus lowest.

Subtracting, in this way, lowest from highest peak in the P3 bounding window, will, in most cases, yield a positive peak-to-peak value. Thus, in our group-level P3 analysis, a comparison against zero is inappropriate, and we require a ‘no-effect’ baseline to compare against. The inclusion of Irrelevant2 trials gives this baseline. Thus, we also calculate an Irrelevant2 peak-to-peak by, in the same way, subtracting out the Irrelevant1 ERP and calculating an Irrelevant2 peak-to-peak value on the Irrelevant2 minus Irrelevant1 difference wave. We then compare Probe peak-to-peak value to Irrelevant2 peak-to-peak value. This contrast is demonstrated in Tables 2 and 3, which show the group-level comparison between the Irrelevant2 and Probe conditions.

**Table 2:** Peak-to-peak P3a sizes for all 15 participants.

	Probe		Irrelevant2	
Part. No.	Fz	Cz	Fz	Cz
1	10.017	7.975	2.998	2.805
2	9.957	6.572	2.081	0.291
3	8.627	5.278	2.867	1.988
4	11.177	8.880	2.488	0.800
5	11.848	7.776	6.340	5.343
6	9.434	17.536	1.564	2.675
7	7.404	7.587	2.870	1.606
8	7.800	7.863	0.942	1.625
9	10.277	10.823	2.975	3.954
10	4.690	2.425	3.066	3.575
11	5.522	2.541	3.625	4.057
12	6.780	8.890	−1.492	0.098
13	9.747	10.263	4.109	4.784
14	6.381	6.304	1.306	2.332
15	9.503	8.589	1.970	2.194

This table shows peak-to-peak differences (relative to Irrelevant1) for both Probe and Irrelevant2 conditions. Note that Probe is consistently larger than Irrelevant2.

**Table 3:** Peak-to-Peak P3b-Pz sizes across the fifteen participants.

Part. No.	Probe	Irrelevant2
1	4.169	4.344
2	6.656	6.225
3	10.145	3.398
4	9.025	2.166
5	12.714	6.231
6	25.840	5.025
7	11.985	3.739
8	22.864	5.423
9	12.148	4.451
10	9.195	4.979
11	6.976	3.581
12	14.704	3.254
13	12.184	4.028
14	4.631	2.500
15	9.657	1.178

Peak-to-peak differences (relative to Irrelevant1) are shown here. Note that Probe is larger than Irrelevant2, for most participants.

### 2.8 First Level: Single dimension randomisation

For each electrode, we undertake a separate first level randomisation; thus, electrodes Fz, Cz and Pz serve as single dimensions. We then perform a second level analysis, which determines a combined significance across these dimensions/electrodes. We discuss these first level randomisations here.

In the previously presented P3a and P3b analysis methods, we have liberally determined optimal parameters for the measure of interest, e.g. inner window placements for peak-to-peak analysis. With standard statistical methods, such post hoc identifications would be suspect and, at the least, subject to prohibitive multiple comparisons correction. Randomisation and the logic of maximal statistics sidesteps this difficulty [35]. Specifically, generating a null hypothesis distribution reflecting the *maximal* value for a particular measure on each random resample, automatically controls for multiple comparisons in parameter selection for this measure. We applied a randomisation procedure in order to determine a participant's null hypothesis distribution. (Note, a trial is effectively a triple, with P3a-Fz, P3a-Cz and P3b-Pz segments. In this way, we maintain the correlations across electrodes within trials.) Before the procedure started, the least number of valid trials between the Probe, Irrelevant1 conditions was determined (valid trials are free of eye blinks and other artifacts); we call this number *m*. This least number of valid trials varied between 41 and 50 (*M*: 46.7, *SD*: 3.39). *m* trials were, then, selected from the Probe condition, and *m* from the Irrelevant1 condition. These selections were performed at random, without replacement.

The randomisation procedure was the same at each electrode (Pz, Fz, Cz); for each it proceeded as follows. First, two vectors (each of size *m*) were randomly populated with the 2×*m* selected trials. Note, under the null hypothesis, Irrelevant1 and Probe trials would be samples from the same distribution - the null distribution - and would thus be exchangeable. Second, a pair of ERPs were generated, one from each vector. One of these ERPs notionally playing the Probe role and the other the Irrelevant1 role. A peak-to-peak difference between the two ERPs was then calculated. The procedure repeated until 1,000 values were obtained; these 1,000 correspond to the null hypothesis distribution.

**Figure 4:**
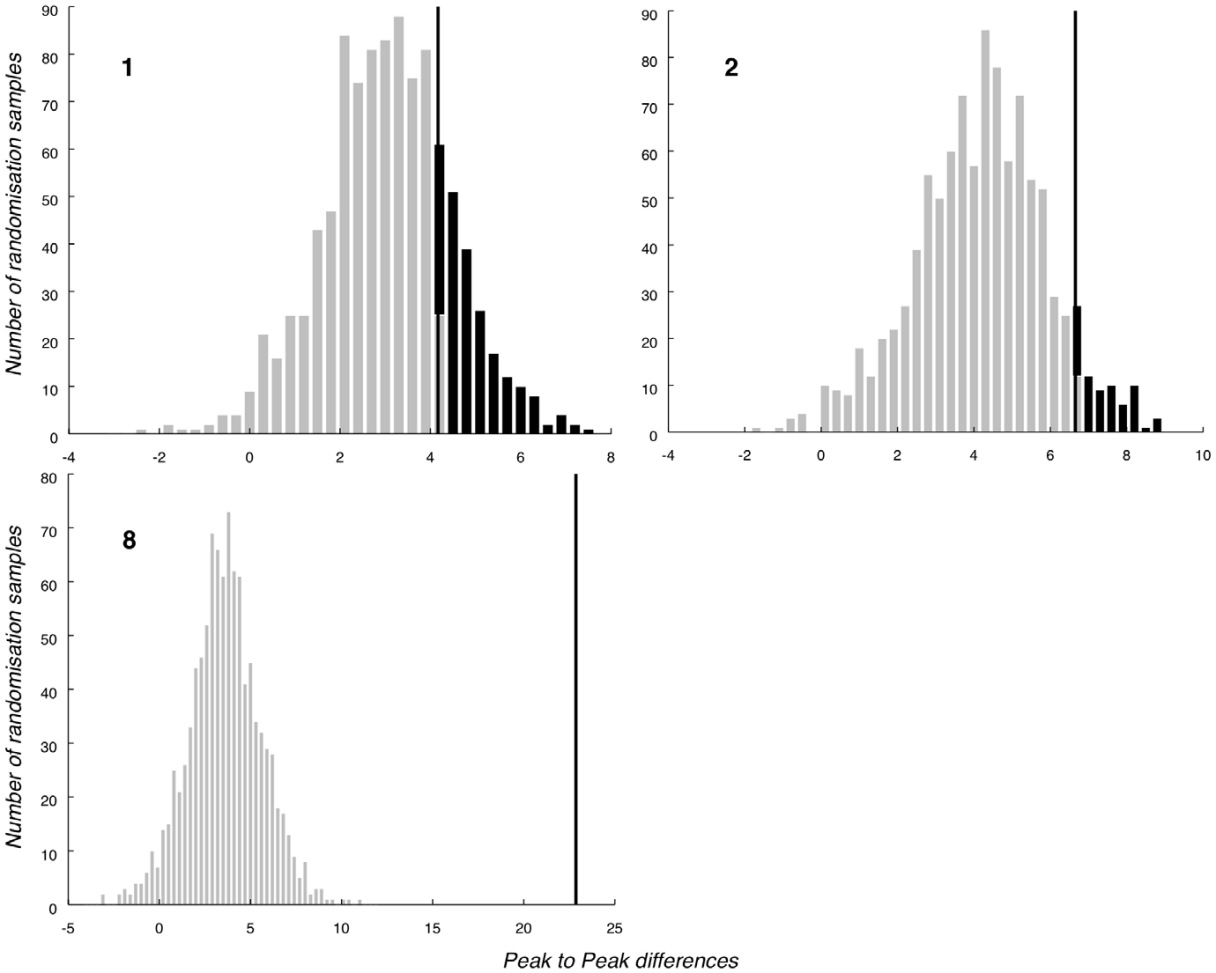
Selection of P3b-Pz null hypothesis distributions. Illustrative P3b randomised null hypothesis distributions for three participants (1, 2 and 8, whose ERPs are shown in Figure 10). The true observed value is marked by a vertical line, with area above that line, which gives p-value, marked. Data from Participant 1, whose P3b effect is weakest (as seen in their ERP) produces a large p-value. On the other hand, the true observed value for Subject 8, the participant whose effect is strongest, falls far outside of the randomised null hypothesis distribution, resulting in a p-value<0.001.

A p-value was determined as follows: the true observed value was obtained from the (true) ERPs of the given participant, as the peak-to-peak of the difference between the (true) Probe and (true) Irrelevant1 conditions. (Note, there was no need to compare to the Irrelevant2 against Irrelevant1 baseline highlighted in section 2.7, since the randomisation distribution plays the role of baseline. For example, in Figure 4 the baselines are the underlying distributions (both black and light grey areas), while the actual (Probe) peak-to-peak measurement is shown by the vertical line (true observed value).) The p-value was then calculated as the number of randomised peak-to-peak values that were greater than the true observed value, divided by 1,000. Since, as previously discussed, we apply this same procedure at the three electrodes (Pz, Fz, Cz), we obtain three, Probe against Irrelevant, p-values.

### 2.9 Second Level: Combined analysis

For each participant, the data from the three single dimension randomisations (P3a-Fz, P3a-Cz and P3b-Pz) described in section 2.8 were used to compute a joint p-value under a Fisher combined probability test. A number of methods for combining different dimensions of statistical significance have been considered [37], [41]. The Fisher method (discussed in Hayasaka and Nichols) treats the different dimensions consistently, since by combining *p-values* of individual dimensions, it automatically normalises into a common comparable measure. A dimension where there are very large (raw) differences between data points would have a disproportionate effect on the combined significance without such normalisation.

To determine a combined p-value for one participant across electrodes (P3a-Cz, P3a-Fz and P3b-Pz), we first calculated 1,000 single dimension p-values, for each electrode. Each such p-value reflects where one data point (denoted *d*), arising from our original random resampling (which was described in section 2.8), sits in its single dimension randomisation distribution. That is, a p-value was obtained by determining the proportion of the 1,000 values present in the single dimension randomisation distribution that were above *d*. This gave us 3,000 p-values: 1,000 for each electrode/dimension, with associations across dimensions, such that data point *i* in the P3a-Fz electrode corresponds to point *i* in the P3a-Cz electrode and point *i* in the P3b electrode (since these three data points were generated from the same random sample). Finally, 1,000 Fisher scores were obtained by using the following formula:
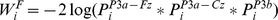
where, *i* ranges over the 1,000 random samples. The key aspect of this formula is that the p-values from single dimensions are multiplied.

Similarly, a Fisher score was calculated on the true observed data point using the same formula. An overall, cross dimension p-value was, then, obtained by calculating how many of the 1,000 random sample Fisher scores were above the true observed Fisher score, and then dividing by 1,000. When calculating Fisher scores, values of p = 0 (which would result in the formula returning infinity) were replaced by the smallest legitimate p-value, 0.001 (1/1,000).

This Fisher method works well with our data. An illustration of this is that, when there is room for p-values to change, i.e. all three single dimension p-values have not hit their minimum value (0.001, with the 1,000 random samples we perform), the combined p-value (after the Fisher procedure) is typically substantially below the average of the three single dimension p-values. As a demonstration of this, see participants 2 and 11 in Table 4. This is because Fisher combining method does a good job of trading significance levels off across dimensions. Specifically, considering the two dimension case to simplify explanation, there are two combinations of p-values that do well. Firstly, a pair of p-values where one is very small (i.e. highly significant) will tend to obtain a small p-value under the Fisher's method, even if the other single dimension p-value is relatively large. This provides a disjunctive element to combining, i.e. a bias towards the minimum of the two p-values. Secondly, a pair of p-values where both are almost significant can yield a significant Fisher combined p-value. This provides a conjunctive element to combining, i.e. a bias towards simultaneously low p-values.

**Table 4:** Single dimension randomisations results and Fisher combined probability scores.

Part. No.	P3a-Fz	P3a-Cz	P3b-Pz	Fisher
1	0.001	0.002	0.208	<0.001
2	0.005	0.093	0.066	0.008
3	<0.001	0.017	<0.001	<0.001
4	<0.001	<0.001	<0.001	<0.001
5	<0.001	0.008	<0.001	<0.001
6	<0.001	<0.001	<0.001	<0.001
7	<0.001	<0.001	<0.001	<0.001
8	<0.001	<0.001	<0.001	<0.001
9	<0.001	<0.001	<0.001	<0.001
10	0.150	0.600	0.001	0.012
11	0.122	0.566	0.010	0.039
12	0.005	<0.001	<0.001	<0.001
13	<0.001	<0.001	0.001	<0.001
14	0.046	0.013	0.199	0.019
15	<0.001	<0.001	<0.001	<0.001

Shown above are p-values obtained from the single dimension randomisations and combined three-dimensional Fisher procedure, for all participants and all conditions. Fisher scores (on which the decision of deception is based) are <0.05 for all participants, resulting in an overall 100% hit rate.

Thus, if the true observed values sit in either of these areas, i.e. overwhelming evidence on one dimension or a lot of evidence on two dimensions, Fisher can generate a p-value below the average of single dimension p-values. The opportunity to benefit in this way, though, is dependent upon the level of correlation between the component dimensions. Specifically, p-values change more under Fisher's method as dimensions become more independent.

We clarify these aspects with a simulated exploration of Fisher's method in Appendix S1 and Figures S1, S2 and S3. However, the upshot of these characteristics for our data is that the benefit of combining P3a-Cz with P3a-Fz is small, since the two electrode dimensions are somewhat correlated. In contrast, combining P3a (either Fz or Cz) and P3b-Pz brings a substantial benefit, since firstly, these dimensions are uncorrelated (see Appendix S2 and Figure S4) and secondly, true observed values do tend to fall in the two areas in which benefit can accrue.

### 2.10 Intrinsic validity of statistical inference

To explore the intrinsic validity of our statistical method and thereby, verify our implementation of it, we undertook a simulation study when the null hypothesis held by construction. In this way, we calculate the method's true false positive rate (i.e. true Type I error rate), which in the limit should equal the alpha level, in our case 0.05. A particular reason for doing this was to confirm the validity of our use of Fisher's method, which may be considered a nonstandard technique. Note, we do not explore in simulation the other criteria for judging our statistical test's worth, i.e. the statistical power (which determines the Type II error rate), since we view our results for real empirically collected data (i.e. the paper's main finding) as evidence of the method's power.

To assess the false positive rate, we ran the analysis on a dataset that could not contain any signal (i.e. was pure “noise”), but that preserved “background” temporal correlations that arise in all EEG time series, independent of any effect being investigated. Thus, we analysed segments locked to distractors in Irrelevant trials, that were free from artefacts (such as eye blinks). Given that the critical item (in this case, Irrelevant1 or Irrelevant2) can appear in position 5 to 10 within the RSVP stream, there are 5 distractors in an Irrelevant trial that appear in the critical range of positions. Selecting 4 segments stimulus-locked to these distractors at random, enabled us to generate single trial segments that are very unlikely to contain signal. Such segments were then added to a single, all segments, pool, from which segments were assigned to fabricated Probe, Fake, Irrelevant1 or Irrelevant2 conditions. This selection was performed fully at random, so that any segment could be assigned with equal probability to any condition. This process resulted in 45 to 67 trials (depending on the number of usable segments for each participant) for each fabricated condition. 1,000 of these datasets (i.e. containing a fabricated Probe, Fake, Irrelevant1 and Irrelevant2) were created and our standard ERP and randomisation analyses were applied on each dataset, resulting in 1,000 P3b-Pz, P3a-Fz and P3a-Cz p-values, which were then used to generate their respective 1,000 p-values under Fisher's method. In the limit, we should find 5% of these Fisher scored p-values to be below 0.05. The proportion of p-values below 0.05 is the intrinsic false positive rate.

### 2.11 Empirical False Positive Rate

The previous section considered the intrinsic false positive rate of our statistical analysis, i.e. the theoretical Type I error rate, which is inherent to the method. Another issue is the false positive rate of our deception detection approach in general, of which the statistical analysis is just one part.

In one respect, the randomisation procedure controls the false positive rate, by explicitly calculating the null hypothesis distribution and deriving a p-value from it; that is, by considering the consequence of interpreting the Probe and Irrelevant as samples from the same distribution. However, the true *empirical* false positive rate is the chance of interpreting a nondeceiving participant as deceiving and that requires considering a situation in which what the experimenter considers to be a Probe in fact really is an Irrelevant. Put another way, our randomisation procedure calculates the false positive rate when the Probe is *hypothetically* treated as an irrelevant, but, because all participants are lying about their identity in our main experiment, the Probe was in fact indeed their real name. But, there remains the possibility that participants behave differently if there really is no condition in which their name is present. For example, it might be that without a Probe to notice, Irrelevants would be more easily seen. This is the question we explore in our empirical false positive rate experiment.

Specifically, we ran our experiment on a control group. We recorded data from 8 participants, who were students at the University of Kent, in the age group 18–22 (*M*: 19.25, *SD*: 1.49). All were paid for participating. All participants were native English speakers and right handed. Six were female, two male. Participants were free from neurological disorders and had normal or corrected-to-normal vision. Only native English speakers participated in the experiment.

We utilised exactly the same stimulus presentation, stimuli and recording apparatus previously highlighted for our main experiment (sections 2.2–2.4). The only difference being that there was no Probe, but rather three Irrelevants: Irrelevant1, Irrelevant2 and Irrelevant3, each selected at random from the set of possible names, without informing the participant of their identity. Thus, their real name never appeared in the experiment. Handling of the Fake was unchanged.

This gave us three identical conditions for each participant: Irrelevant1, Irrelevant2 and Irrelevant3, each of which comprised three sets of trials - one for each electrode: Fz, Cz and Pz. The three Irrelevants at each electrode yielded six pairwise comparisons, since there are six permutations of three, e.g. (Irrelevant1, Irrelevant2), (Irrelevant1, Irrelevant3), (Irrelevant2, Irrelevant3), (Irrelevant2, Irrelevant1), etc. We ran our statistical analysis on each such pair, with the first in the pair playing the (notional) Probe role and the second the Irrelevant role. Across the eight participants, this gave us 48 data sets, each comprising notional Probe at Fz, Cz and Pz and Irrelevant at Fz, Cz and Pz. We analysed each data set with single dimension randomisations for Fz, Cz and Pz and then a Fisher combining. This gave us 48 tests of an empirically-enforced null hypothesis. From this we can determine an approximate empirical false positive rate.

### 2.12 Early trials - Late trials comparison

As previously discussed in section 1.3, this analysis sought to determine whether the repeated presentation of Irrelevant trials could influence their electrophysiological response. Accordingly, for each participant, the first half of the Irrelevant2 trials (arising early in the experiment) was assigned to an Early Irrelevant2 condition, whereas the remaining half (arising late) was assigned to a Late Irrelevant2 condition. (If frequency of presentation increased the ability to perceive, Late Irrelevant2 should show more evidence of a P3). ERPs were generated from each of Early and Late Irrelevant2, with one for each channel of interest (Fz, Cz and Pz), resulting in 6 ERPs in total per participant.

An Early-Late (peak-to-peak) P3 analysis was then performed at Pz, Fz and Cz. For each of these, the ERP generated from the Early Irrelevant2 condition was subtracted from the ERP generated from the Late Irrelevant2 condition. A peak-to-peak measurement was obtained from the resulting difference wave, in the same procedure as described in section 2.7. This resulted in a peak-to-peak value for each participant. It was not, though, appropriate to compare the resulting values against 0, since, as previously highlighted, the peak-to-peak values are more likely to be positive than negative under the null hypothesis. Rather, we need a baseline difference to compare against in which no P3 would be present; we selected to compare Irrelevant1 Even and Odd trials with Early - Late Irrelevant2s.

The index number for each Irrelevant1 trial (which were in chronological order) was used to determine whether trials were to be assigned to the Even or Odd condition, so that trial 1 was assigned to the Odd condition, trial 2 to Even and so on. ERPs were then generated for both Even and Odd Irrelevant1 for all electrodes, and an Even - Odd difference wave was calculated at each electrode for each participant. No temporal effects or, indeed, P3 effects at all could be present in this Even-Odd difference wave, i.e. it was an appropriate baseline. Even - Odd peak-to-peak values were then calculated from these difference waves. Corresponding peak-to-peak values were then compared between Early - Late Irrelevant2 and Even-Odd Irrelevant1 in a paired t-test.

### 2.13 Latency difference analysis

As previously discussed, in order to verify a difference in temporal features between Fake and Probe, we performed a latency contrast. The P3a latency difference was assessed at the Fz electrode, while the P3b latency difference was assessed at Pz. For both analyses (P3a and P3b), a Fake - Probe latency difference was assessed by comparing the latencies of the two grand average ERPs. Although similar, the parameters changed between P3a and P3b analysis. They consisted in a bounding window (*b*) and an inner window (*w*). For the P3a, *b* started at 150 ms and ended at 400 ms from target onset. For the P3b, these values were changed respectively to 300 ms and 1000 ms. The inner window (*w*) was 50 ms for the P3a and 100 ms for the P3b. This difference reflects broadness disparities between characteristic P3as and P3bs.

The latency of the grand average P3 was determined by sliding an inner window of width *w* across the time range *b* and finding the inner window placement with the maximal average voltage. The start of that time window was taken as the latency of the P3 for the given grand average ERP. The two latencies (Fake - Probe) were then subtracted from each other, resulting in a single latency difference measurement.

To assess statistical significance of latency difference between Fake and Probe, a randomisation analysis was applied on all trials in the experiment, i.e. a fixed effects analysis. The procedure started by creating two pools: one for the Probe condition and the other for the Fake condition, each containing all such trials for all participants. The two pools were then combined into a single “Both Conditions” pool. Then, repeatedly, two disjoint sets of *m* trials were randomly selected from Both Conditions, without replacement (*m* being the least number of total trials in either the Fake or Probe conditions). Two surrogate “grand average” ERPs were then generated from the two sets, and the latency difference between them was calculated, as just discussed. The procedure repeated 1,000 times, resulting in a 1,000 latency differences (which comprise the estimated null hypothesis distribution). A p-value was computed by first calculating how many of these differences were above the true-observed grand average ERP difference, and then dividing this number by 1,000.

## Results

### 3.1 Basic group-level effects

#### 3.1.1 Early fronto-central component

We observe a clear fronto-central full oscillation cycle, which is large and early for the Probe, medium-sized and slightly later for the Fake and absent for Irrelevant1 and Irrelevant2, as shown in the grand averages for Fz and Cz (Figure 2). This component is initially positive, with a following damped negative deflection. As justified further in the *Discussion* section, we interpret this as a P3a. Our key group-level P3a statistical test is a paired t-test of a peak-to-peak analysis of Probe P3a and Irrelevant2 P3a across participants. Peak-to-peak values for Probe and Irrelevant2 across participants are shown in Table 2. This analysis was separately applied at Fz and Cz and both paired t-tests were highly significant Fz: p<0.0001, 95% confidence interval (of difference from Irrelevant2) was 4.9079∼7.2854; Cz: p = 0.0001, 95% confidence interval was 3.2201∼7.6030.

#### 3.1.2 P3b component

Grand average ERPs for the four conditions at the Pz electrode are presented in Figure 1. Positive deflections in the identified P3b region are clearly evident for Fake and Probe. The P3b elicited by the Fake name has the largest amplitude. This is as one would expect, since detection of Fakes is the explicitly performed task. The Probe, though, also generates a robust group-level P3b, although, it is somewhat smaller and earlier than the Fake P3b. As for the P3a analysis, peak-to-peak values for both Probe and Irrelevant2 were compared and are shown in Table 3. A paired t-test of Probe against Irrelevant2 was computed, resulting in a very significant difference between the two conditions: p = 0.0002, 95% confidence interval 4.3136∼10.6694.

### 3.2 Early-late Analysis

It is clear from our analyses that if participants detect Irrelevants it is rare enough not to generate a robust P3a or P3b. However, as previously discussed in section 2.12, to obtain further confidence that Irrelevants are not detected, we compared Early and Late (after they had been frequently presented) Irrelevant2s to Even and Odd Irrelevant1s. We then performed our P3 analyses and compared the results between Early - Late (Irrelevant2s) and Even - Odd (Irrelevant1s).

For the P3b, a paired t-test was performed to establish whether there was a significant difference between Early - Late set and Even - Odd. The test failed to reject the null hypothesis that Early - Late and Even - Odd are samples from the same distribution: p = 0.2251 and 95% confidence interval −3.2792 to 0.8412. For the P3a, the paired t-test Early - Late against Even - Odd yielded p = 0.4484, with 95% confidence interval −0.9691 to 2.0767 (Fz electrode) and p = 0.6404, with 95% confidence interval −1.3335 to 2.0972 (Cz electrode).

**Figure 5:**
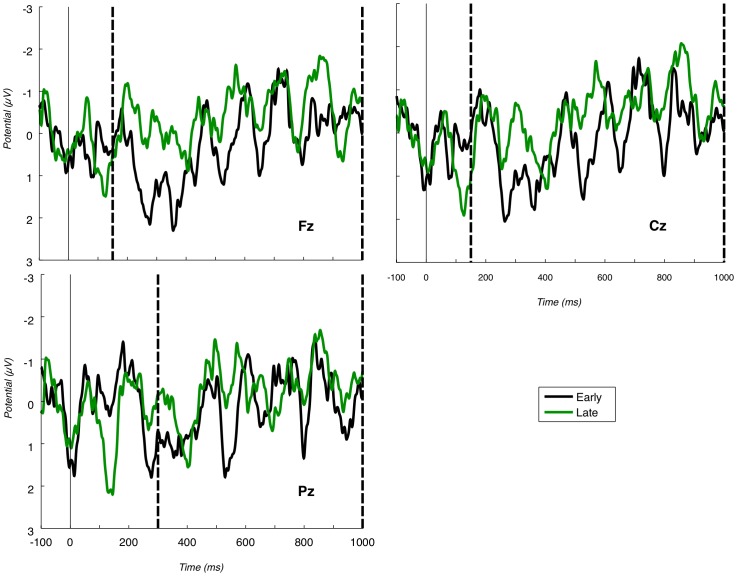
Early vs. Late grand averages for all channels. Grand average ERPs for Early vs. Late. The ‘Early’ trace is the grand average for the first half (in chronological order) of Irrelevant2 trials that each participant was presented, while the ‘Late’ trace is the grand average for the second half of Irrelevant2 trials. There is no clear indication of a difference between the two conditions (especially, the amplitudes of the ‘Late’ traces are not greater than ‘Early’ ones). Vertical dashed lines demarcate the relevant P3 bounding window.

These tests provide no evidence of a difference between Early and Late Irrelevants, as also suggested by the corresponding grand average ERPs (certainly, Late Irrelevant does not seem bigger than Early Irrelevant, Figure 5).

### 3.3 Latency difference

**Figure 6:**
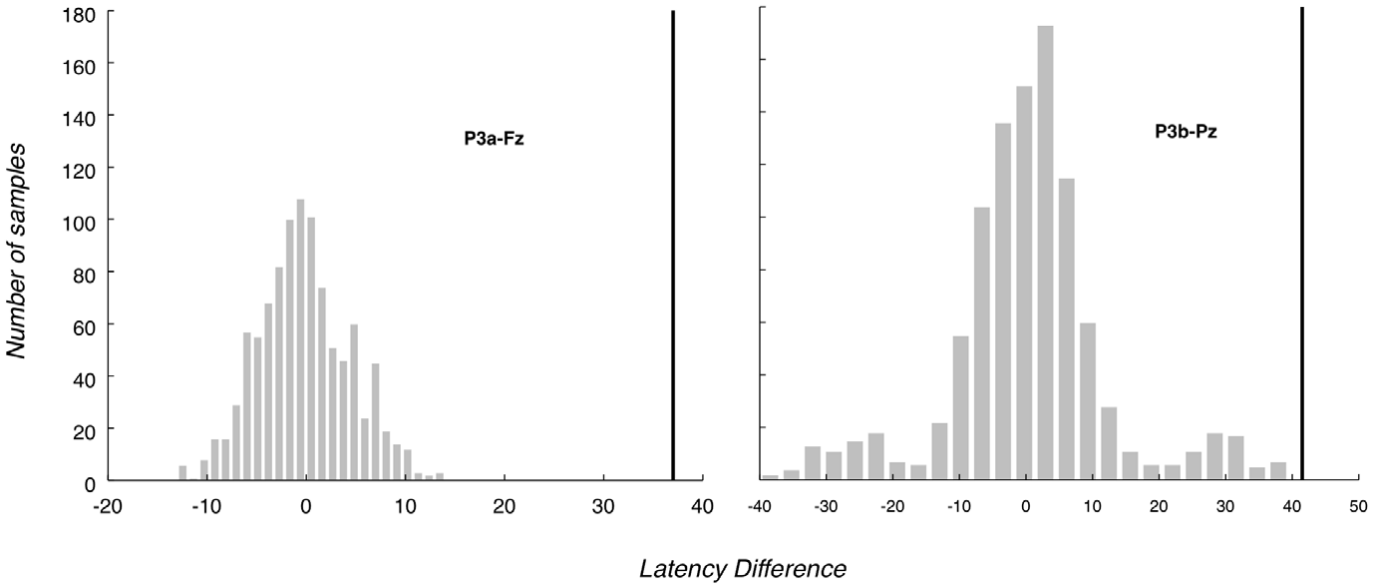
Latency difference null hypothesis distributions. Randomisation inferred null hypothesis distributions aggregated across all participants for latency differences between Fake and Probe. Black vertical lines mark true observed value and p-value region. P3a calculated on Fz electrode and P3b at Pz. These show that the difference in latency between Fake and Probe is significant.

The Figure 6 distributions show the randomised latency differences between Fake and Probe. The black line shows the true observed grand average difference. This suggests that the P3a and P3b p-values are no more than 0.001, since no randomised data points were above the grand average latency difference. For both electrodes, there is then evidence of a latency difference between Fake and Probe, so that the P3 elicited by Probe trials is earlier than the P3 elicited by Fake trials.

### 3.4 Analysis by individual

#### 3.4.1 Intrinsic Validity of Randomisation

As previously discussed, see section 2.10, to confirm the intrinsic validity of our statistical method, we assess its true false positive rate (i.e. the Type I error rate) in simulation. This involves determining the likelihood of obtaining a p-value below a particular alpha level (in our case 0.05) when the data analysed do not contain any effect, i.e. for which the null hypothesis is true.

**Figure 7:**
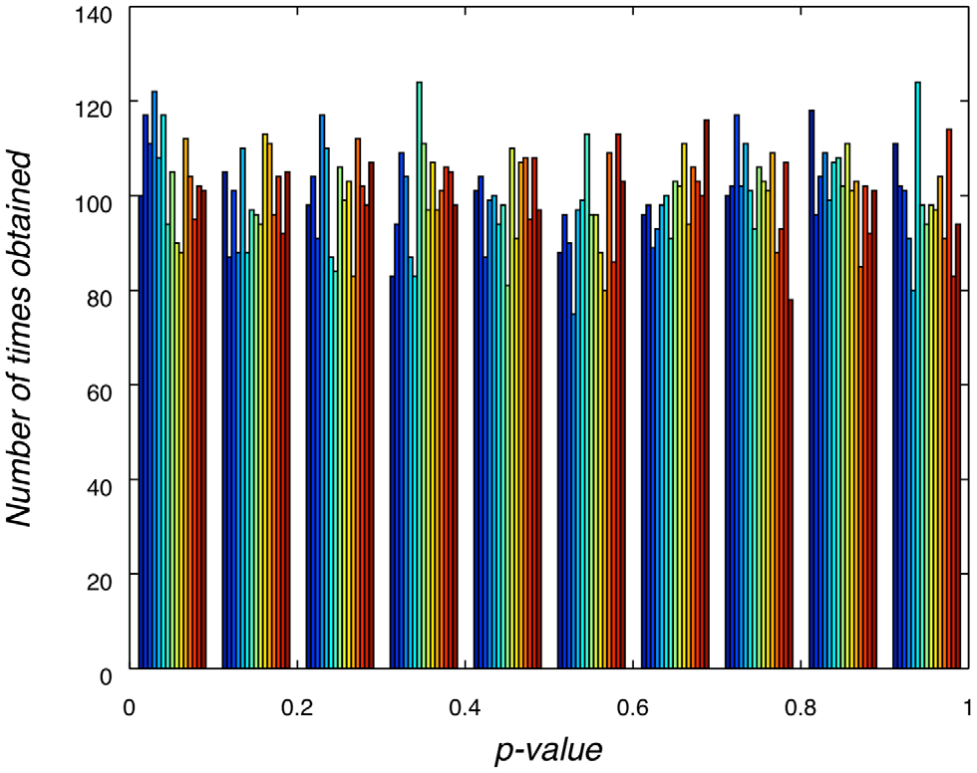
Distribution of p-values obtained from the intrinsic validity test. Distribution of p-values combined under Fisher's method obtained from the intrinsic validity false alarm testing procedure. The 15 different bars in each bin represent the data obtained from the 15 participants. As expected, the distribution of p-values is uniform.

The results of our analysis applied to such “noise” data (i.e. trials which were not expected to contain any signal) are shown in Table 5. As expected, all average p-values for all single and combined randomizations are close to 0.5. Also, the “False Positive Rates” for all single and combined randomizations are close to 0.05, showing that the number of false alarms generated by our analysis method is no larger than the statistically acceptable standard. Figure 7 depicts the distribution of the 1,000 p-values under Fisher's method for each one of the 15 participants. As expected, the distribution is uniform.

**Table 5:** Results obtained from the intrinsic false alarm testing procedure.

	P3a-Fz		P3a-Cz		P3b-Pz		FISHER	
Part. No.	*FP* Rate	Avg p	*FP* Rate	Avg p	*FP* Rate	Avg p	*FP* Rate	Avg p
1	0.048	0.512	0.051	0.514	0.052	0.499	0.057	0.512
2	0.056	0.493	0.058	0.499	0.059	0.504	0.062	0.497
3	0.054	0.499	0.053	0.501	0.054	0.500	0.054	0.501
4	0.055	0.493	0.068	0.486	0.046	0.506	0.058	0.488
5	0.046	0.491	0.054	0.488	0.065	0.485	0.061	0.485
6	0.050	0.523	0.052	0.523	0.054	0.493	0.061	0.518
7	0.043	0.514	0.042	0.511	0.042	0.490	0.045	0.505
8	0.053	0.498	0.048	0.502	0.054	0.503	0.048	0.498
9	0.050	0.507	0.037	0.515	0.040	0.510	0.039	0.513
10	0.054	0.491	0.045	0.497	0.043	0.504	0.049	0.498
11	0.051	0.504	0.053	0.499	0.053	0.490	0.050	0.499
12	0.061	0.501	0.059	0.488	0.054	0.489	0.069	0.486
13	0.051	0.503	0.054	0.512	0.049	0.504	0.047	0.505
14	0.040	0.492	0.041	0.494	0.043	0.509	0.037	0.496
15	0.052	0.488	0.049	0.485	0.054	0.508	0.053	0.492
**AVG**	**0.051**	**0.501**	**0.051**	**0.501**	**0.051**	**0.500**	**0.053**	**0.500**

*FP* Rates indicate the frequency with which a p-value <0.05 was obtained during a run (out of 1,000 runs, for each participant). As expected, *FP* Rates are close to a value of 0.05.

The average p-values obtained across the 1,000 runs are around 0.5, which is as expected (last row). This table was generated using the method described in section 2.10.

#### 3.4.2 P3a

**Figure 8:**
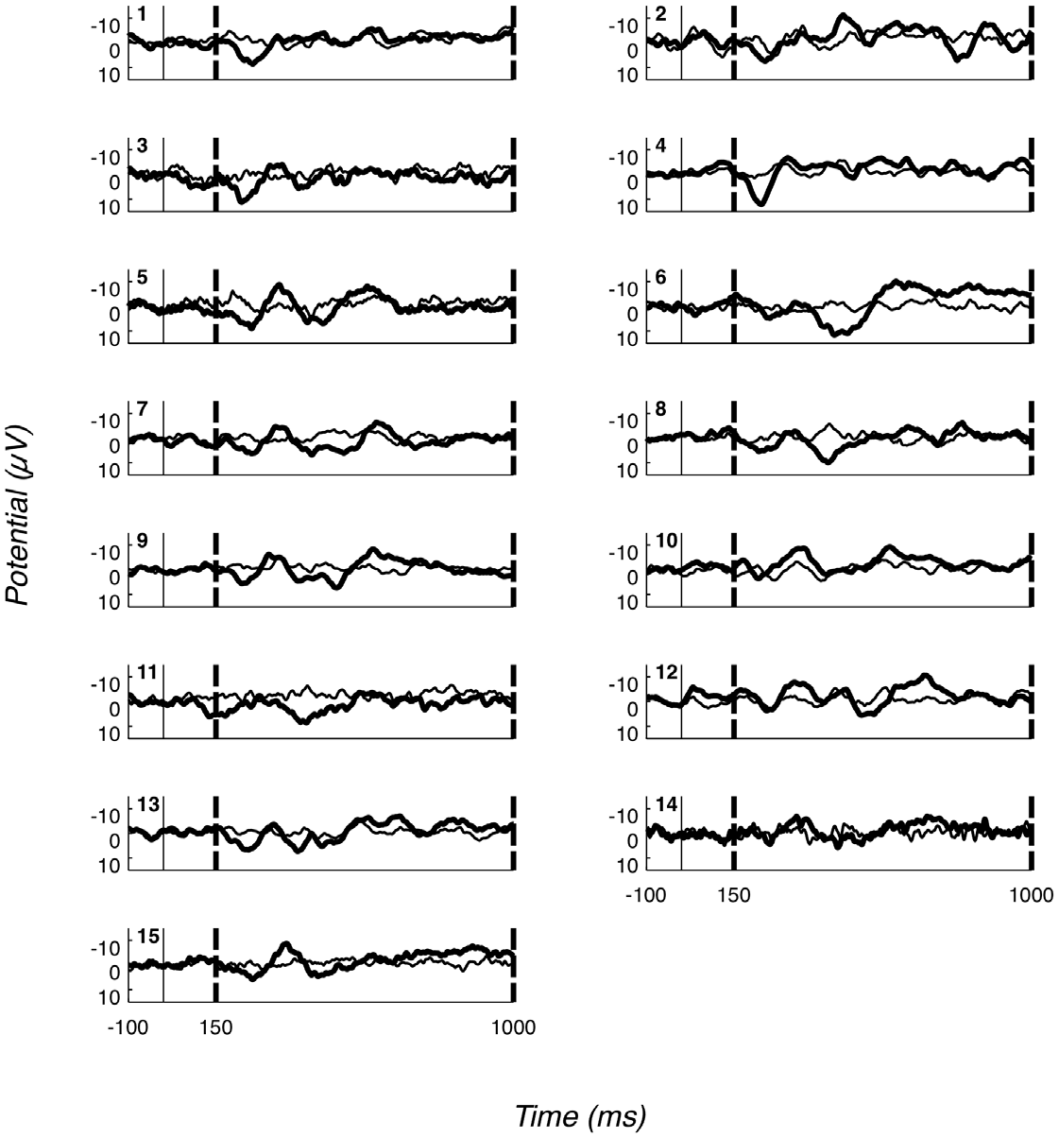
Fz ERPs for all participants. Fz ERPs for all participants; positive down. Dashed vertical lines represent the P3a bounding window. The bold line is the ERP for the Probe condition, while the thinner line is the ERP for the Irrelevant1 condition. The number on the top left of each plot indicates the participant. The P3a effect (Probe more positive early and/or more negative late than Irrelevant1) is identifiable for each participant.

**Figure 9:**
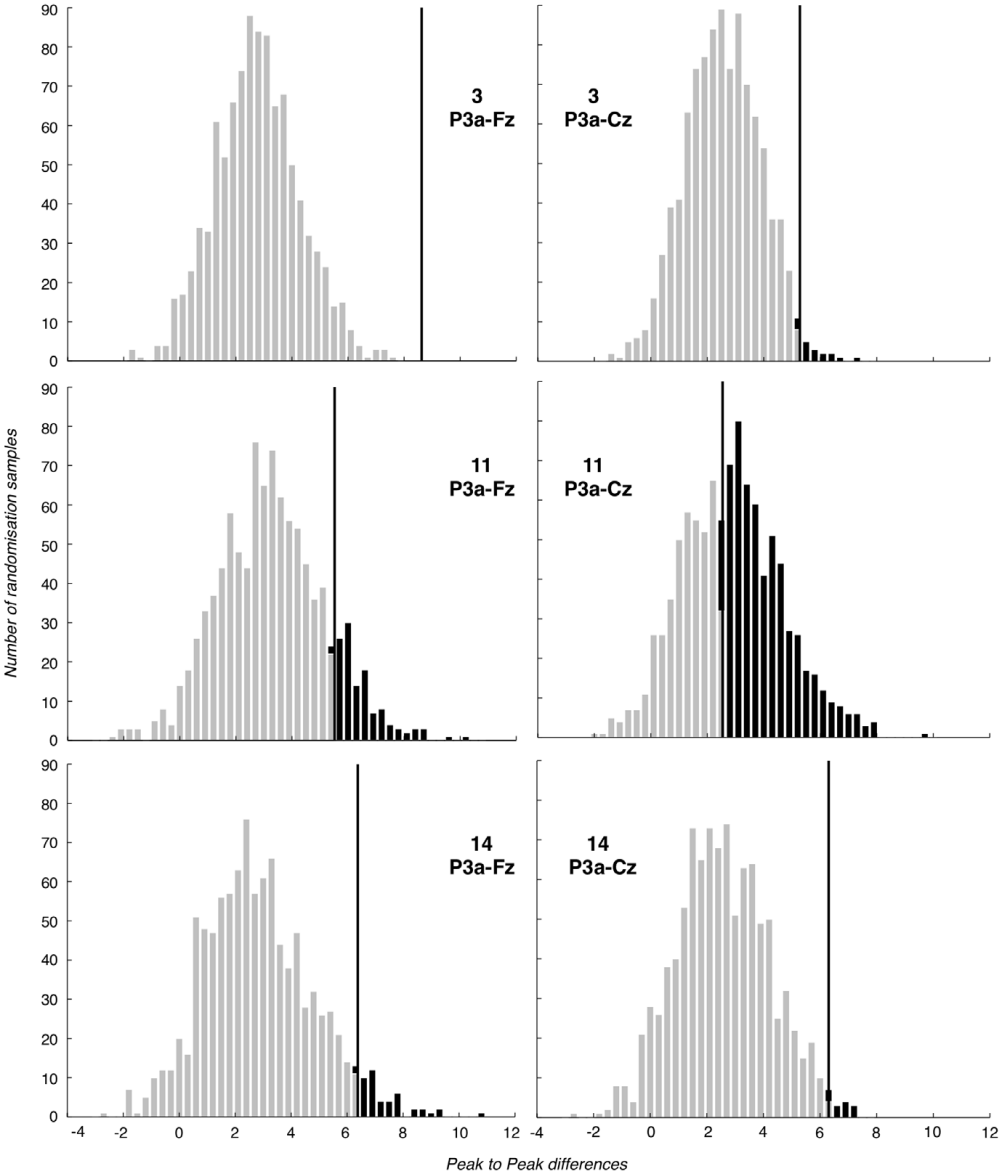
Selection of P3a-Fz and P3a-Cz null hypothesis distributions. Fz and Cz P3a (peak-to-peak) null hypothesis distributions for three representative participants (participants 3, 11 and 14). True observed value and Type I error region are marked in black.

The consistency and robustness of the P3a component across participants can be seen in Figure 8, where participant (average) ERPs for Probe trials at Fz are shown, with P3a bounding region marked by dashed vertical lines. For almost all of the 15 participants, a full-oscillation cycle can be seen. The relative size of positive deflection to following negative deflection varies by participant, but a peak-to-peak difference is clear for all. To illustrate our methods, Figure 9 presents P3a null hypothesis distributions generated through randomisation at Fz and Cz, for three representative participants. In particular, one of these (P11) has the weakest P3a component, while the other two (P3 and P14) are typical. As can be seen, the mean peak-to-peak P3a difference under the null hypothesis is participant-dependent, but generally between 1 and 4. This reflects the mean value of a peak-to-peak analysis when no component is present.

Table 4 presents the per-individual p-values arising from P3a peak-to-peak randomization analysis at Fz and Cz. As should be clear, this test proves extremely effective. In addition do also note, the smallest p-value we can obtain with a thousand resamplings is 0.001. However, the exact veridical p-values for many of these participants are likely to be significantly smaller. For example, for participant 3 at Fz in Figure 9, the nearest null hypothesis value remains far from the true observed value, suggesting that many more iterations of a thousand resamplings could be performed before a null hypothesis value extreme relative to the true observed value would arise.

#### 3.4.3 P3b

**Figure 10:**
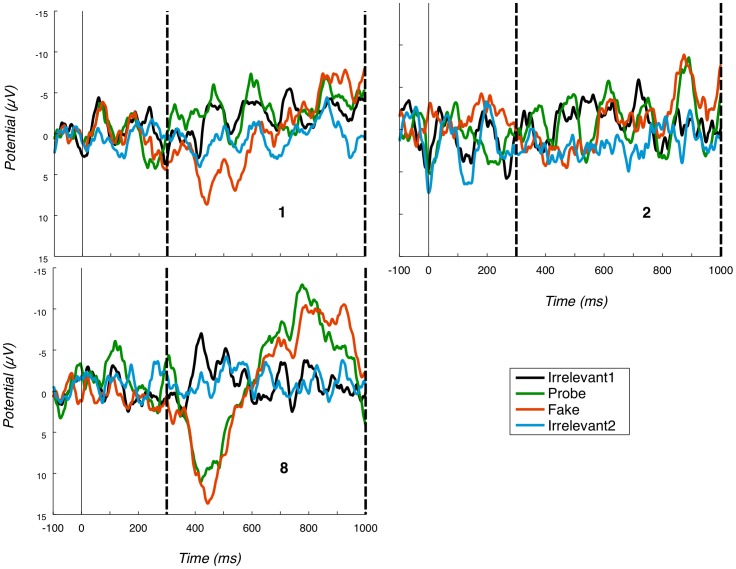
Selection of Pz ERPs. Pz ERPs for all conditions for the participants considered in Figure 4 (1, 2 and 8). Positive is plotted down. Vertical dashed lines mark the P3b bounding window.

While we have obtained a robust group-level P3b for Probe against Irrelevant1, there are individual differences in this measure. As a reflection of this, Figure 10 presents Pz ERPs for participants 1, 2 and 8 (which are chosen to illustrate boundary conditions in our analysis, rather than for their absolute typicality). The first of these, participant 1, exhibits a Probe waveform without a clear positivity, and which does not differ much from the Irrelevant1 condition (marked with a thin black line). Thus, the P3b bounding window contains no evidence of deception for participant 1. While extremely noisy, the Pz ERP for participant 2 does suggest a weak effect on the strength of the peak-to-peak analysis employed. During the P3b bounding window, there is a slight positivity for Probe around 525 ms, followed by a negativity at around 900 ms (note that the Fake, at least to some extent, follows a similar pattern). However, the Irrelevant1 condition does not show such clear peaks, resulting in a significant Probe - Irrelevant1 peak-to-peak difference. In contrast, participant 8's Probe has clear and very high (in absolute value) peaks. The corresponding randomization tests for these three participants are presented in Figure 4. The participant 1 distribution contains no evidence to reject the null hypothesis, while the participant 2 distribution is approaching a weak rejection and the participant 8 distribution suggests a highly significant rejection. Per-individual significance tests are summarised in Table 4.

### 3.5 Combined analysis

**Figure 11:**
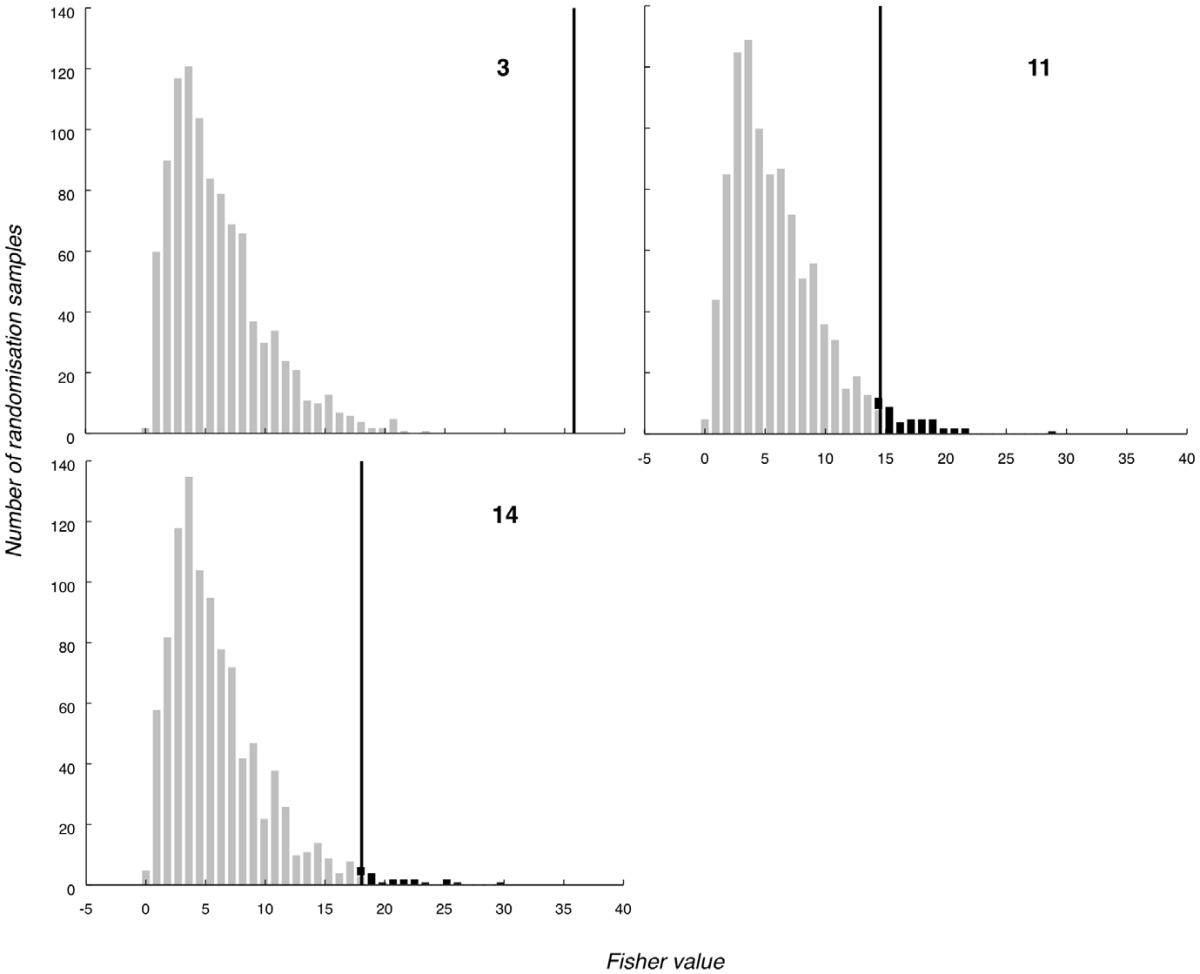
Selection of Fisher score distributions. Fisher (P3a-Fz, P3a-Cz, P3b-Pz combined) null hypothesis distributions for three participants (subjects 3, 11 and 14; see also Figure 9) using Fisher's method. Black vertical lines mark true observed Fisher scores and p-value regions.

Table 4 also shows the p-values obtained for each participant in combined 3-dimensional inference, using Fisher's method. For most participants (11 out of 15), the p-value was smaller than 0.001; that is, when the three dimensions (P3-Fz, P3-Cz and P3b) were weighed together, there were no null hypothesis data points above the true observed value, clearly indicating presence of those participants' real name. Participant 11 has the largest p-value, but still well below a 0.05 alpha level, again successfully detecting “own-name” occurrence. Figure 11 depicts the distribution of Fisher values for the same three participants we considered the P3a for, with a black line showing the Fisher value of the true observed grand average data point.

### 3.6 Empirical False Positive Rate

As previously discussed, we are also interested in the false positive (i.e. Type I error) rate of our overall deception detection approach, over and above the intrinsic false positive rate of our statistical inference method. Out of the 48 null data sets collected, 3 yielded significant p-values, see Table 6. This is a little higher than the theoretical (inference method) false positive rate, i.e. the alpha level, which implies a false positive rate of 5%. We discuss this finding in section 4.3.

**Table 6:** Empirical false alarm test results.

Part. No.	P1	P2	P3	P4	P5	P6
1	0.301	0.744	0.837	0.980	0.191	0.118
2	*0.019	*<0.001	0.755	0.174	0.600	0.416
3	0.055	0.085	0.944	0.743	0.400	0.058
4	0.745	0.575	0.547	0.850	0.570	0.707
5	0.559	0.944	0.072	0.842	0.064	0.528
6	0.109	0.859	0.060	0.995	*0.016	0.078
7	0.221	0.661	0.083	0.544	0.433	0.173
8	0.754	0.394	0.698	0.743	0.239	0.476

Shown above are the results for the empirical false alarm testing procedure that was applied to our control group. The outcomes that were significant at an alpha level of p<0.05 are indicated by an asterisk. Three false alarms are present. The columns refer to all possible permutations, given that the three Irrelevant conditions were assigned a different role of Probe, Irrelevant1 and Irrelevant2 in each permutation. This was described in section 2.11.

## Discussion

### 4.1 Summary

We have highlighted EEG-marked subliminal salience search (eegSSS) as a means to apply theoretical work on rapid serial visual presentation (RSVP) and accompanying electrophysiological correlates of salient stimulus detection/identification. We have then illustrated this technique in the context of detecting identity deception. Specifically, we demonstrated robust EEG differences between trials in which participants behaviourally lie about their identity and trials containing no salient item. At an individual-level (which is statistically demanding), we were able to demonstrate a selective brain response to their real name (the Probe), at an alpha level of 0.05, for all the 15 participants. Furthermore, we were able to demonstrate, at an alpha-level of 0.05, a selective response to their name (the Probe) for 13 (respectively 12) participants out of 15 for the P3a-Fz (respectively P3a-Cz). In addition, the average p-value for the P3a-Fz (respectively P3a-Cz) was 0.02 (respectively 0.09). Then, we combined P3a and P3b analyses using a three-dimensional Fisher combined probability procedure, to obtain an average combined analysis p-value of 0.006 and 100% detection of a distinct response to the Probe at an alpha level of 0.05 (73% at an alpha level of 0.01). In addition, some of these p-values are likely to be substantially larger than their actual values, since we restricted ourselves to 1,000 random resamplings and some true observed values remain far from the nearest null hypothesis data point. Finally, we have now replicated this experiment a number of times, providing what is effectively a large sample size when accumulating across replications and the effectiveness of the method at the individual-level carries over to this larger sample size (Bowman H, Filetti M, Alsufyani A, Janssen D, Su L, et al. (2013) Countering countermeasures: detecting identity lies by detecting conscious breakthrough. Under Submission. Unpublished data).

### 4.2 Deception detecting as salience detection

In what sense is the proposed method truly detecting deceit? It is certainly not the case that eegSSS is directly revealing the occurrence of a lie or, indeed, observing a brain signal that is unique to a lie. Rather, it is detecting the occurrence of a perceptual event, initiated by the brain's detection of a stimulus that is salient, where that salience could be due, for example, to familiarity or affective charge. Indeed, stimuli upon which a lie is made are often very salient, particularly if criminal guilt or innocence is at stake.

This said, the Guilty Knowledge (or Concealed Information) Test, e.g. [28], requires a number of Probes (perhaps 5 or 6), each of which should only be “known” to the guilty. It has been argued [46]–[48] that the requirement for so many, may mean that some Probes would necessarily be incidental to the crime (e.g. the colour of the carpet at the crime scene) and would not carry exceptional affective charge. Lie detection in this context effectively becomes a familiarity test. Although probably more weakly, familiarity alone should still be sufficient to mark a stimulus out as salient in RSVP subliminal search, which in turn would cause breakthrough into consciousness, thereby, generating a P3. In contrast, stimuli that are guilt-irrelevant, task-irrelevant and, also unfamiliar, such as Irrelevants, should remain subliminal and, thus, not generate a P3. In this way, through appropriate choice of stimuli, our proposal would specialise a salience-detection system into a deception-detection system. This said, the absolute effectiveness of our approach beyond own-name stimuli (which clearly carry a particularly pronounced salience) remains for future investigation, especially when those stimuli are only incidentally significant [46].

Clearly, instructing freely volunteered participants to lie with respect to their name, with no legal sanction at stake, is artificial (although proof of identity is, indeed, a common forensic concern) and surely does not replicate the affect-ladden relevance of true guilt determining identity tests. Thus, one might expect that the electrophysiological response to identity would be even bigger when guilt is at stake. In this sense, our study can be seen as a conservative test and our success in per-individual analysis bodes well for more realistic instantiations of the technique.

The critical demonstration in our study is that intrinsic salience, which lays at the heart of lie detecting, can induce P3 patterns that are statistically detectable within individuals. In particular, we instructed participants to look for their Fake name, respond yes to its presence, no to their real name (the Probe) and no to streams perceived as containing neither Fake nor Probe. Thus, the same action was associated with Probe and Irrelevant stimuli. However, only the former yielded P3 components.

Moving specifically to our claim to be detecting an intrinsic salience response, it is true that we are not absolutely certain of the task-set employed by participants. That is, we do not know how our instructions at the beginning of the experiment are implemented as a cognitive task to perform by a participant during the experiment. The ERP-patterns will certainly be sensitive to strategic enforcement of such a task set. However, the effects we have obtained would seem to yield robust detection of an intrinsic salience response whatever particular cognitive task strategy was at play. There are two obvious explicit task-sets that participants could be employing: 1. Probes like Irrelevants or 2. Probes like Fakes; we consider these in turn.

#### 1. Probes like Irrelevants

Since participants are instructed to respond identically to Probes and Irrelevants, i.e. say no, it could be that the task-set employed is ‘look for Fakes, in order to respond yes and, as an effective default, respond no to Fake absence’. In other words, in respect of the task set, Probes and Irrelevants would be treated identically, since they both reflect Fake absence. However, if such a strategy were being employed, the clear and substantial difference in ERP-signature between Probe and Irrelevant could only be explained as a differential intrinsic salience response to the Probe.

#### 2. Probes like Fakes

As highlighted earlier, the alternative explicit task-set would be that, somehow, participants employ a strategy of looking for Probes in order that they can be sure of lying to them, i.e. saying no. If this were the case, then the task-set would be ‘look for both Fakes and Probes’. Hypothesising such a task-set, though, would not subvert our proposal re. intrinsic salience, since the Fake and the Probe were also distinguishable by ERP-signature. Specifically, (as demonstrated in section 3.3) the Probe P3b-component was earlier and also smaller than the Fake P3b and more significantly, the P3a for Probe was earlier and also larger than for Fakes. Thus, even if one were to believe that participants were explicitly searching for the Probe, there was nonetheless still a Probe-specific pattern (especially for the P3a), which one would naturally believe was a distinctive marker of intrinsic salience over and above task salience.

Thus, our contention is that whichever explicit task strategies participants might be employing, the ERP patterns we obtained would still suggest a differentiable response to intrinsic salience.

With regard to countermeasures, it would certainly seem that a strategy of artificially increasing the salience of Irrelevant stimuli is, at least significantly, subverted. One way of putting this is that, in the case of intrinsic nonsalience, and putting the possibility of chance perceptions (which we discuss shortly) aside, the subconscious brain can only enhance representations of stimuli (to enable pop-out), if the conscious mind can direct it to ‘look’ for such stimuli. However, the conscious mind can only know to direct the subconscious mind at Irrelevant stimuli if they have already been “seen” through subconscious selection; that is, the Irrelevant can only be “seen” once it has been “seen” (and is thus “known”). In broader terms, our use of target detection during RSVP forces participants to rely on subconscious cognitive mechanisms, which are fundamentally less amenable to top-down control. As empirical confirmation of this intuition, in a forthcoming paper (Bowman H, Filetti M, Alsufyani A, Janssen D, Su L, et al. (2013) Countering countermeasures: detecting identity lies by detecting conscious breakthrough. Under Submission. Unpublished data), we show that the method remains effective even when we tell participants how it works and give them an explicit countermeasure strategy to apply, such as, attempting to see Irrelevants on the basis of their frequent presentation and then, once seen, count the number of times it is seen.

A remaining countermeasure that might be attempted is to ‘determinedly’ enforce task focus on the Fake, to the extent that the intrinsic salience of the Probe is somehow displaced from cognitive focus. It is true that our current study has not definitively ruled out such a countermeasure. However, we believe it unlikely that such a strategy could fully eradicate the differential ERP-signature we obtained for Probes. That is, identity is intrinsically salient in the extreme and it would be very surprising if cognitive control could “reach down” and volitionally counteract such salience. Indeed, such a capacity for cognitive control to nullify intrinsic salience would seem to be ruled out by a whole spectrum of psychological disorders, where inherent concern-relevant stimuli and thoughts dysfunctionally intrude into the conscious mind's cognitive thread. Another way of putting this is that if such volitional control of the brain response to intrinsic salience were possible, many psychological disorders, e.g. posttraumatic stress disorder, anxiety, addictive behaviours and even, depression, could be ‘easily’ treated by sufferers simply applying conscious effort to exclude condition-relevant stimuli and thoughts (indeed, directed forgetting experiments [49] explicitly demonstrate an inability to consciously exclude even rather unsalient memories).

In fact, as suggested by such psychological disorders, one might expect that the very process of attempting to exclude a response to a certain stimulus would place that stimulus more fully in the focus of the perceptual system. Furthermore, as we have argued, the distinctiveness of the ERP-pattern for Probe relative to Irrelevant and Fake, does seem to suggest a response to Probe that is associated with its intrinsic salience. The notion that task-set can, through volitional effort, specifically counteract intrinsic salience to the point of excluding its electrophysiological response, seems unlikely.

Another concern that might be raised is that our approach could be susceptible to the trivially simple countermeasure of attending away from the stream. Such attending away could involve overt shifts of attention, i.e. in which eyes move, or covert shifts in which the attentional spotlight moves in the visual field, without eye movement. Such shifts would, though, be detectable by an eye tracker for overt gaze shifts and via the Steady State Visual Evoked Response (SSVEP) for both overt and covert shifts of attention. The SSVEP is generated from the regular presentation format of an RSVP stream, which when attended, sets up an oscillation in the brain at the stream's frequency. Absence of power and inter-trial coherence at the presentation frequency at posterior electrodes could, thus, serve as a marker of participant noncompliance. Indeed, in a similar fashion, it would seem that the method is robust against a spectrum of similar countermeasures, such as, modulation of level of alertness, muscle tension, eye movements or eye blinks. The critical point here being that inawareness of the Irrelevants means none of these can be applied selectively to the Irrelevant. As a result, the only strategy that can be used to confound the method is to *continuously* apply the confounding activity, e.g. attempt to permanently “phase out” and hold a low alertness state. Such sustained applications of a confound strategy should be observable as a reduced SSVEP and Fake P3.

### 4.3 Type I Error Rate

We demonstrated that our statistical inference method is intrinsically valid, i.e. has a Type I error rate equal to the alpha level; see sections 2.10 and 3.4.1. In addition, we explored the empirical Type I error rate, i.e. the deception detection method as a whole's false positive rate; see sections 2.11 and 3.6. In this empirical check, we did find a false positive very close to the alpha level: 3 data sets yielded significant p-values out of 48, the alpha level being 0.05.

### 4.4 Limitations

Strictly, we have demonstrated a method to detect concealed identity information. What then, is the potential of eegSSS for lie detecting in general? As in fact with all current lie detection approaches, there are limitations to the proposed method and the technique needs to be applied with care. Perhaps most significantly, Probe and Irrelevant stimuli need to be selected very carefully. Firstly, Irrelevant stimuli must not be salient to the participant. For example, if by chance, the Irrelevant was familiar, then a P3 for Irrelevant could be generated, confounding the Probe minus Irrelevant comparison. Such an eventuality should, though, be apparent from inspection of the Irrelevant ERP, enabling a new Irrelevant to be selected and a new eegSSS run.

The more pernicious error in stimulus selection would be use of a Probe that is familiar to a non-guilty suspect. One would hope that the guilty would find a crime-relevant stimulus more salient than the non-guilty, thereby, generating larger P3(a and/or b) responses, but presence of even weak Probe P3s might yield a false positive attribution of guilt to the innocent. Thus, users of an eegSSS lie detector would need to assiduously avoid priming the Probe during pre-lie detection questioning and ensure that the selected Probe has not appeared in the media.

A further issue to consider is the absolute subliminality of Irrelevant stimuli. In particular, in a noisy biological system such as the brain, some stream stimuli will be perceived, by what, within the context of current scientific understanding, would be attributed to chance. Clearly, the frequency of Irrelevant stimuli makes them candidates for such chance perceptions. However, in our experiment, Irrelevant perception was rare enough not to generate a P3a or P3b, which could confound the distinctiveness of the ERP signature of Probe stimuli. The absolute subliminality of Irrelevant stimuli and whether any semblance of a usable countermeasure can be devised on this basis is a topic for further consideration. In particular, even faster presentation rates could be employed if Irrelevants were, by chance observed, or one could insert many Irrelevants, to the point that a participant's capacity to notice all as frequent and accordingly, hold all in task focus, would become prohibitively difficult.

The key question, though, that will govern whether the proposed approach can be generalised to broader lie detection domains, is the extent to which the dramatic differences in Probe and Irrelevant ERP signatures, is specific to own-name stimuli. To be clear, such specificity of the method would not render it valueless. In particular, questions of own identity are of great forensic relevance. For example, detection of malingering or feigning amnesia [40] is an important topic for neuropsychologists. In this context, deception detection would indeed focus on autobiographical information, such as own name. The autobiographical salience of such information should ensure a large ERP response of the kind seen here.

The application of eegSSS to broader lie detection contexts, such as the Guilty Knowledge test, where stimuli of only incidental salience are probed, is less certain and awaits further empirical studies.

### 4.5 Naming and nature of P3a

An important issue to consider is our identification of the early fronto-central component as a P3a. Certainly, the majority of RSVP P3 work has focused on the P3b, and there have been few, if any, identifications of P3as in the RSVP context. However, this may be because ours is the first ERP study in which intrinsic (not strictly task-prescribed) salience has been explored with RSVP. While we acknowledge some room for debate, we have ascribed the name P3a for a number of reasons. Firstly, although full scalp topography is not available with the small number of electrodes we have deployed, the component observed, as found with the classic odd-ball P3a, is largest frontally with a gradually reducing signature with posterior progression. Secondly, while we observed the Probe fronto-central component peak very early (positively around 240 ms), the corresponding Fake component peaks positively around 280 ms, which is a latency more in keeping with the classic odd-ball P3a. It would certainly seem that the Probe and Fake fronto-central deflections in our data can be viewed as the same kind of component. Consequently, if one identifies the Fake component as a P3a, then one necessarily must make the same attribution to the Probe component and explain the latter's early positive peak by the undeniably exquisite personal salience of one's own name. Thirdly, our fronto-central component is largest in the Probe condition, where, as we have argued, intrinsic salience is most significant. This fits with the interpretation of the classic P3a.

Specifically, although there remains disagreement, it is classically argued that the cognitive antecedents of the P3a and the P3b differ in respect of task salience. That is, in classic oddball tasks [39], [50], [51], the P3b is typically largest for a task-relevant oddball. However, the P3a is largest for an oddball that is outside the task-set; accordingly, ‘Novelty P3’ is often used interchangeably with P3a. Although not an identical characterisation, the association in our experiment of our fronto-central component with intrinsic salience does mesh with the Novelty P3 notion. That is, in our experiment, the P3b was clearly largest for the Fake, which was, effectively, task-prescribed as the target, while the P3a was largest and earliest for the Probe, which we would argue is more strongly associated with (task-incidental) intrinsic salience.

Fourthly, we observe a full oscillation cycle, especially for the Probe. However, classic P3a's in the odd-ball literature are typically just half cycle positivities. Our observation of a full cycle pattern in the Probe condition, may simply be attributable to the extreme salience of one's own name. In particular, it may be that at the single trial level, the P3a always manifests as a strong positivity followed by a weaker negativity. However, with less salient and, perhaps more diverse, target stimulus sets there may be considerably more latency jitter in the P3a across trials. This would overlay positive and negative deflections across the latter portion of the grand average component, where the greater size of the positive deflection would attenuate the negative deflection. Indeed, this overlay effect may be so pronounced that in the grand average, the component is observed as a broad medium-amplitude positivity. Indeed, in our data, the following negativity is reduced, if not absent, in the (weaker) Fake condition grand average. Confirmation of this hypothesis awaits application of single trial analysis techniques such as inter trial coherence and phase sorting [3].

### 4.6 Oddballs

While the distinction between a novelty-driven and task-driven P3 can inform the distinction between our P3a and P3b components, other aspects of the classic oddball conceptualisation of the P3 do not naturally carry over to our findings. In particular, in terms of stimulus presentation, the most marked Oddballs in our experiments are, in fact, the distractors: each of which appears just once or twice in the entire experiment. However, distractors fail to generate any P3 at all, either P3a or P3b. The classic oddball theory would though predict that P3s should be largest for distractors. Furthermore, Irrelevants, Probes and Fakes are equally frequent in our experiment, however, they generate dramatically different P3 patterns. Specifically, for Irrelevants, P3s are completely absent; while for Probes, the P3a is large and early, and the P3b is largeish and early; while for Fakes, the P3a is small and late, and the P3b is large and lateish.

In addition, if one thought the P3 would decrease in size with increased expectation for its occurrence, one would predict that Probe P3s would get smaller over the course of the experiment. We have found no evidence for such a decrease. All these aspects suggest that a mechanism other than experimental frequency/predictability modulates the P3s we observe.

For us, this other mechanism is salience detection. Robustly, we get clear and large P3s for salient stimuli, i.e. Probe and Fake, and effectively P3 absence for nonsalient stimuli, i.e. Irrelevants and distractors.

### 4.7 Analysis techniques

This paper has brought a number of ‘non-standard’ EEG analysis techniques to bear. In particular, we advocate the broader use of Monte Carlo resampling techniques, such as randomisation and bootstrapping. These have previously been used in ERP studies where individual-specific statistical inference is sought [28]. However, such methods could also prove valuable for group-level analyses. In particular, they have the virtue of being non-parametric, liberating statistical inference from a number of well foundedness criteria.

A domain in which Monte Carlo resampling could be particularly valuable is initial exploratory studies where the literature offers few, if any, a priori precedents for component latency, width, polarity or size. Strictly speaking, ERP components observed in such exploratory studies are subject to a prohibitive correction for multiple comparisons since, for example, the number of possible component time windows is massive. Random resampling methods largely bypass this multiple comparisons problem by directly generating a distribution of *maximal* values for a key measure, such as component length, under null hypothesis assumptions. The true observed value for this measure can then be compared to the null hypothesis distribution, generating a robust estimate of Type I error probability and without need for Bonferroni correction.

### 4.8 Applications of EEG-marked subliminal salience search

While our focus in this paper has been on deception detection, we do believe that eegSSS is a broadly applicable method. In addition to such forensic applications, example uses include, an image triage system [26]; a brainwave acknowledgement system in cockpit interfaces [15]; an independent brain-computer interface [25], [52], [53] an information-retrieval system and a means of stimulus-rich information presentation [27]. We discuss a number of these applications in more detail.

#### Independent brain-computer interface

An *independent* brain-computer interface (BCI) enables patients with no control over any muscles to interact with computers. Such interaction is arguably where true brain-computer interfaces come to the fore, since more efficient, more peripheral, interaction methods, such as eye-tracking, are ineffective. Since stimuli are presented at fixation, an RSVP-based BCI would automatically be independent. Such a method could, for example, present streams of letters, with participants' task being to count the number of times they see the letter they wish to select. Combined with P3 detection, such a format could be an alternative to the Donchin matrix P3 speller, which may be dependent upon gaze [54]. Indeed, whether requiring eye movements, the Donchin speller certainly requires shifts of covert attention, which are likely to be fatiguing. An RSVP BCI is independent of both overt and covert attention shifts.

#### Detecting consumer preferences

eegSSS could be used in the marketing arena as a means to infer consumer preferences and, arguably, specifically ‘implicit’ preferences. Thus, streams containing product alternatives, perhaps as images, could be presented while consumer preferences are determined via P3 detection.

#### Information retrieval

The capacity to efficiently and accurately search stored data is a hallmark of the modern information technology age. However, some data types are fundamentally more difficult to classify and thus search; images being an example. An alternative is for users to scan stored items themselves, while attempting to match target properties, e.g. “school class photograph in which my friend John had long hair”. Due to its high information bandwidth, RSVP is an obvious presentation format for such search.

#### Stimulus-rich information presentation

The high identification accuracy associated with RSVP, suggests that the majority of stimuli presented are processed to the level of comparison with salience templates. Indeed, it has been demonstrated that even targets missed in an RSVP stream can semantically prime subsequent stimuli [55], [56]. Thus, it may be possible for users to extract meaning, perhaps of a highly schematic form [16], from across an entire RSVP presentation. Indeed, this has been shown for RSVP presentation of sentences [57]. A possible application of such a method would be to attempt amelioration of anterograde amnesia by presenting images of a patient's daily experiences to them in RSVP format.

## Supporting Information

Appendix S1 **Fisher's method in simulation.**
(PDF)

Appendix S2 **Fisher's method: our data.**
(PDF)

Figure S1 **Scatter plot of an uncorrelated two dimensional Gaussian.** Scatter plot representing simulated data from a two dimensional, uncorrelated Gaussian. Each data point in the plot is marked by the difference between the average of the two p-values and the p-value obtained through Fisher's method. The amount of such differences is indicated by the bar on the right. For instance, a value of −0.1 implies that a specific data point has benefitted from the Fisher p-value being 0.1 below the average p-value of the two single dimensions. The wing regions, in which Fisher p-values are much lower than average p-values, are indicated by the letter A. The two thick lines represent the mean of each dimension, while the two thin lines represent the 0.05 threshold for each dimension. The dashed line represents the 0.05 threshold for Fisher's method. Therefore, the data points that fall within the small triangular region (indicated by the letter B) are not significant in either of their single dimensions. However, their combined Fisher score is significant. See also figures S2 and S3.
(TIFF)

Figure S2 **Scatter plot of a slightly correlated two dimensional Gaussian.** Scatter plot representing simulated data from a two dimensional Gaussian, whose two dimensions are slightly correlated (*R* = 0.4). Note that when compared to uncorrelated data (Figure S1), the wing regions (A) contain less data points. Also, the small triangular region (B) is smaller than the one found in the uncorrelated data plot.
(TIFF)

Figure S3 **Scatter plot of a strongly correlated two dimensional Gaussian.** Scatter plot representing simulated data from a two dimensional Gaussian, whose two dimensions are highly correlated (*R* = 0.8). Note that the wing regions (A) are much smaller than the ones found in data which contain no correlation (Figure S1) or a low correlation (Figure S2). The small triangular region (B) is almost non-existent, suggesting that applying the Fisher's method to correlated data does not bring benefit.
(TIFF)

Figure S4 **Scatter plot of P3b-Pz and P3a-Fz obtained from Participant 11.** Scatter plot generated from our randomisation procedure when applied to one of our participants (Participant 11). The true observed value calculated by applying our peak-to-peak procedure on the participant's ERP is indicated on the plot by a black square. Only P3a-Fz and P3b-Pz are shown, P3a-Cz is not, so that this figure can be easily compared to the previous randomly generated scatter plots (Figures S1, S2 and S3). Note that this plot is similar to the uncorrelated data plot (Figure S1). Also note that there is an implicit relationship between dimensions under our randomisation procedure, since each randomised sample is a triplet (see section 2.8); that is, the same trials contribute to the P3a-Fz, P3a-Cz and P3b-Pz on each random sample. For this reason, the fact that the two dimensions shown here are not correlated is not induced by our randomisation procedure.
(TIFF)
