# Supplementary material for: Subliminal Salience Search Illustrated: EEG Identity and Deception Detection on the Fringe of Awareness
Source: PLoS One. 2013 Jan 23;8(1):e54258. doi: 10.1371/journal.pone.0054258 (PMC3553137; doi:10.1371/journal.pone.0054258)
Supplement: Appendix S1 — Fisher's method in simulation. (PDF) [file pone.0054258.s001.pdf]

Fisher’s method in simulation

To illustrate our implementation of Fisher’s combined probability test and how it can generate a significance gain, we present simulated two dimensional data. (We in fact have three dimensions, P3a-Fz, P3a-Cz and P3b-Pz, but the logic arising from considering two dimensions, will generalise to three). We sample randomly from a two dimensional Gaussian, for which both dimensions have zero mean and a standard deviation of one. So, the marginal distributions on each dimension are z distributions. But, we vary the correlation between the two dimensions.

We apply a Fisher combining procedure to this simulated data. That is, we generate a thousand points (each a pair of values) and determine their p-value on each dimension, which is easily done, since marginal distributions are z distributions. Thus, we obtain a thousand pairs of p-values. We, then, apply Fisher’s method to these pairs, to give us a thousand Fisher values, one for each pair. We call these thousand values, a Fisher distribution. We then calculate a p-value for each Fisher value, by seeing where it sits in the Fisher distribution.

The point of interest is how each pair of single dimension p-values changes when mapped to a Fisher p-value. This is what we plot in Figures S1, S2 and S2 for different correlations between dimensions. Each data point in the two dimensional distributions is marked by the difference in p-value between the average of the two single dimension p-values and the Fisher p-value of the two dimensions. Our interpretation is that data points with p-values that go down (compared to the average) benefit under Fisher and those for which p-values go up suffer.

These simulations clearly show that the amount of p-value change (from average of single dimensions to Fisher) increases as the dimensions become less correlated. Indeed, if each data point had exactly the same p-value on both (i.e. perfectly correlated) dimensions, Fisher would have no effect on p-values.

This increase in p-value change is evident in each plot, since as the interdimensional correlation decreases, the number of points which benefit from Fisher combining decreases.

These plots (especially those with low correlations between dimensions) show the regions where p-values decrease under Fisher's procedure. In section 2.9, we highlighted a disjunctive and a conjunctive aspect to when the Fisher can increase significance. The former of these is evident in the wing regions (one very low p-value and the other relatively high), in which Fisher-induced p-values go down and the induced Fisher 0.05 threshold becomes close to perpendicular to the axis on which it has a low p-value. These regions are indicated with the letter 'A' in Figures S1 S2 and S3). Mathematically, this disjunctive aspect arises since multiplying a very small probability by a large one gives a very small probability (e.g. 0.96x0.04=0.038, while 0.5x0.5=0.25, even though the average of 0.96 and 0.04 is the same as for 0.5 and 0.5); very small probabilities generate large Fisher combined scores.

The latter, conjunctive aspect, is evident in the data points in the small triangular region (indicated with the letter 'B'), which are outside both single dimension thresholds, but inside the Fisher-induced threshold. This conjunctive aspect is again due to multiplication of probabilities, which mathematically drives Fisher's method. Specifically, the product of two low, but not significant, probabilities yields a probability that is small relative to many probability pairs in which one probability is

significant (e.g. 0.06x0.06=0.0036, while 0.02x0.2=0.004) and critically, because of interdimension independence, many significant p-values are paired with nonsignificant, and, in fact, far from significant p-values.

The Fisher procedure trades disjunctive and conjunctive aspects off against one another, providing a compromise between the two. Put another way, Fisher's method reflects the perspective that both dimensions being close to their 0.05 thresholds (but neither quite there) or one being well below its threshold is very unlikely to arise unless there is a significant effect present.

Thus, if a true observed value falls into either of these (benefit) null hypothesis regions, p-values can reduce and the likelihood that this will happen will increase as individual dimensions become less correlated.
