# Supplementary material for: Subliminal Salience Search Illustrated: EEG Identity and Deception Detection on the Fringe of Awareness
Source: PLoS One. 2013 Jan 23;8(1):e54258. doi: 10.1371/journal.pone.0054258 (PMC3553137; doi:10.1371/journal.pone.0054258)
Supplement: Appendix S2 — Fisher's method: our data. (PDF) [file pone.0054258.s002.pdf]

Fisher's method: our data

We now extrapolate these ideas to our data. Importantly, P3a-Fz and P3a-Cz are relatively strongly correlated; that is random samples with a large P3a-Fz peak-to-peak value tend to also have a large P3a-Cz value. This is to be expected as the P3a component bleeds into both electrodes. As a result, there is only a weak benefit from Fisher combining P3a-Cz and P3a-Fz.

In contrast, as is evident from Figure S4, for participant 11, P3a-Fz is relatively independent of P3b-Pz (and the same is true between P3a-Cz and P3b-Pz). Consequently, the two regions of p-value benefit, disjunctive and conjunctive, are clearly evident, i.e. p-value benefit to Fisher combining when either one p-value is very very small (and the other is much less small), or if both p-values are small (but neither quite significant). True observed values falling in either of these regions will benefit from Fisher combining. The true observed value for this participant is also shown and it is well within the Fisher-induced threshold. Scatter plots of this kind, with P3a-Fz and P3b-Pz's somewhat independent are typical of the participant patterns we obtain.
