# Supplementary figures and images for: Subliminal Salience Search Illustrated: EEG Identity and Deception Detection on the Fringe of Awareness
Source: PLoS One. 2013 Jan 23;8(1):e54258. doi: 10.1371/journal.pone.0054258 (PMC3553137; doi:10.1371/journal.pone.0054258)

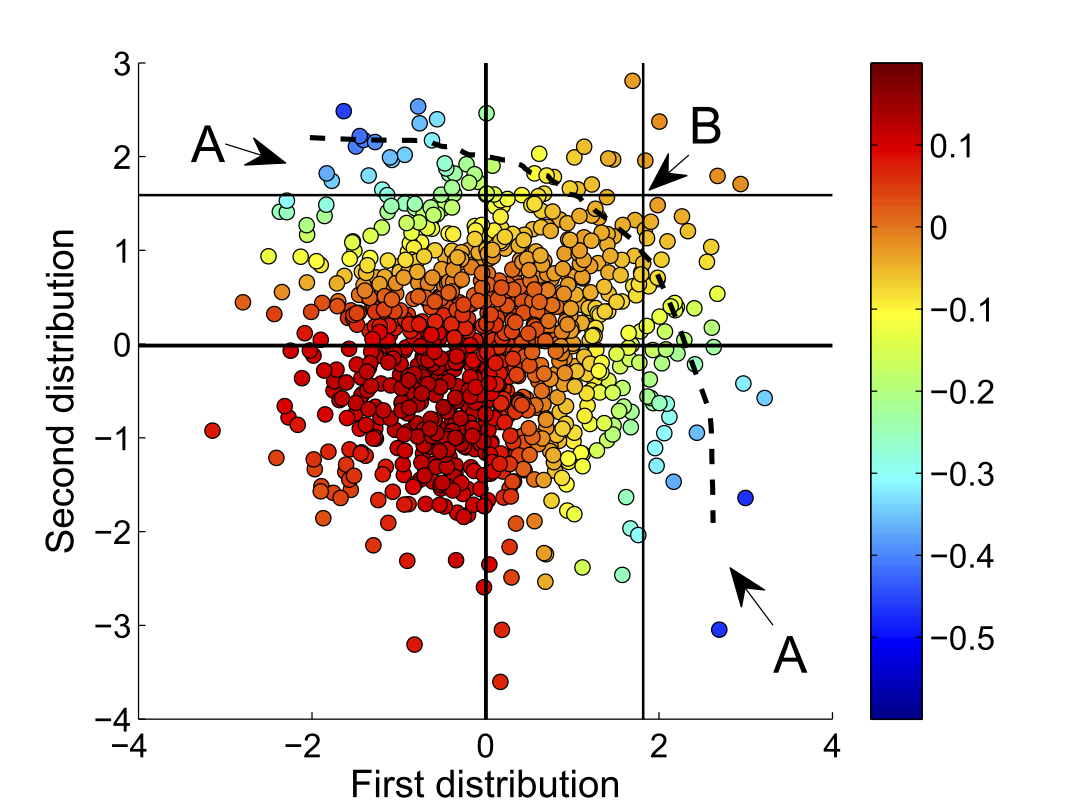

Supplement: Figure S1 — Scatter plot of an uncorrelated two dimensional Gaussian. Scatter plot representing simulated data from a two dimensional, uncorrelated Gaussian. Each data point in the plot is marked by the difference between the average of the two p-values and the p-value obtained through Fisher's method. The amount of such differences is indicated by the bar on the right. For instance, a value of −0.1 implies that a specific data point has benefitted from the Fisher p-value being 0.1 below the average p-value of the two single dimensions. The wing regions, in which Fisher p-values are much lower than average p-values, are indicated by the letter A. The two thick lines represent the mean of each dimension, while the two thin lines represent the 0.05 threshold for each dimension. The dashed line represents the 0.05 threshold for Fisher's method. Therefore, the data points that fall within the small triangular region (indicated by the letter B) are not significant in either of their single dimensions. However, their combined Fisher score is significant. See also figures S2 and S3. (TIFF) [file pone.0054258.s003.tiff]

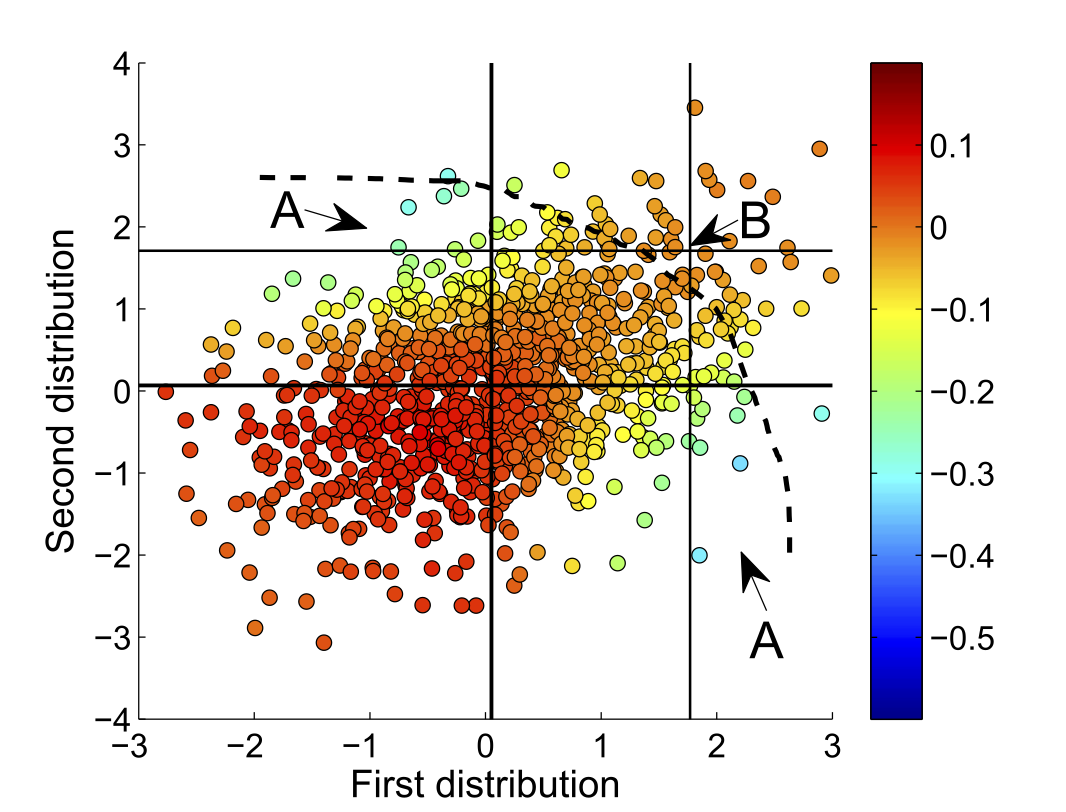

Supplement: Figure S2 — Scatter plot of a slightly correlated two dimensional Gaussian. Scatter plot representing simulated data from a two dimensional Gaussian, whose two dimensions are slightly correlated (R = 0.4). Note that when compared to uncorrelated data (Figure S1), the wing regions (A) contain less data points. Also, the small triangular region (B) is smaller than the one found in the uncorrelated data plot. (TIFF) [file pone.0054258.s004.tiff]

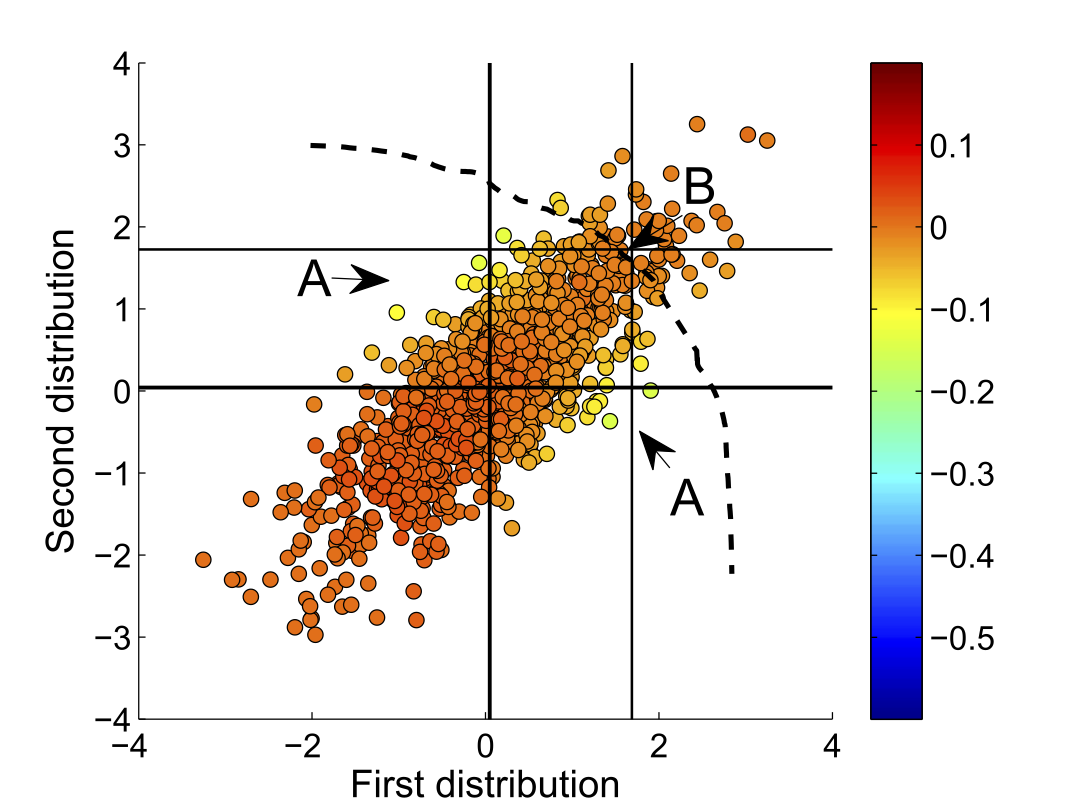

Supplement: Figure S3 — Scatter plot of a strongly correlated two dimensional Gaussian. Scatter plot representing simulated data from a two dimensional Gaussian, whose two dimensions are highly correlated (R = 0.8). Note that the wing regions (A) are much smaller than the ones found in data which contain no correlation (Figure S1) or a low correlation (Figure S2). The small triangular region (B) is almost non-existent, suggesting that applying the Fisher's method to correlated data does not bring benefit. (TIFF) [file pone.0054258.s005.tiff]

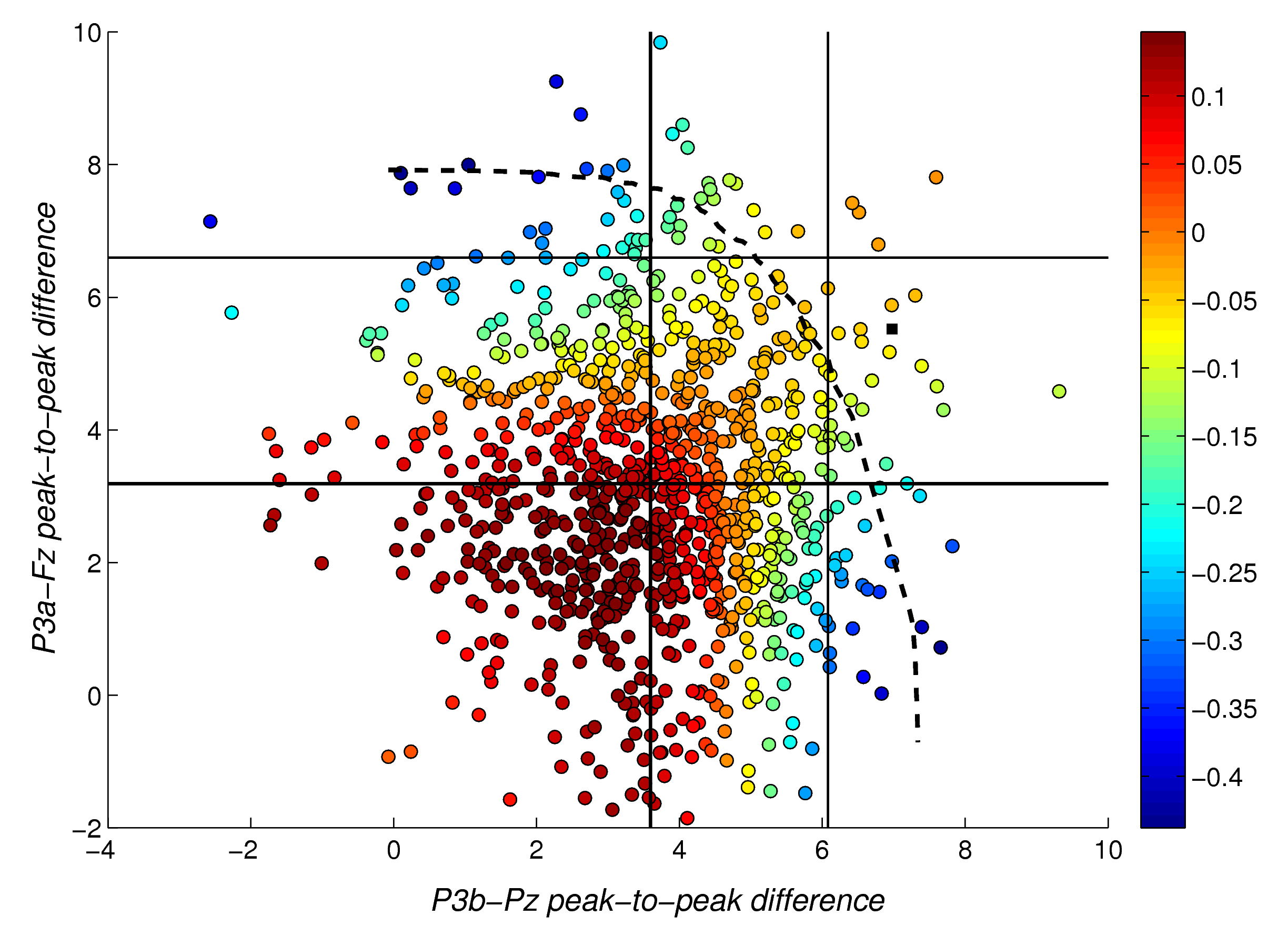

Supplement: Figure S4 — Scatter plot of P3b-Pz and P3a-Fz obtained from Participant 11. Scatter plot generated from our randomisation procedure when applied to one of our participants (Participant 11). The true observed value calculated by applying our peak-to-peak procedure on the participant's ERP is indicated on the plot by a black square. Only P3a-Fz and P3b-Pz are shown, P3a-Cz is not, so that this figure can be easily compared to the previous randomly generated scatter plots (Figures S1, S2 and S3). Note that this plot is similar to the uncorrelated data plot (Figure S1). Also note that there is an implicit relationship between dimensions under our randomisation procedure, since each randomised sample is a triplet (see section 2.8); that is, the same trials contribute to the P3a-Fz, P3a-Cz and P3b-Pz on each random sample. For this reason, the fact that the two dimensions shown here are not correlated is not induced by our randomisation procedure. (TIFF) [file pone.0054258.s006.tiff]
